# Receptor for advanced glycation end products aggravates cognitive deficits in type 2 diabetes through binding of C‐terminal AAs 2‐5 to mitogen‐activated protein kinase kinase 3 (MKK3) and facilitation of MEKK3‐MKK3‐p38 module assembly

**DOI:** 10.1111/acel.13543

**Published:** 2022-01-26

**Authors:** Xiao‐Yan Zhou, Chang‐Jiang Ying, Bin Hu, Yu‐Sheng Zhang, Tian Gan, Yan‐Dong Zhu, Nan Wang, An‐An Li, Yuan‐Jian Song

**Affiliations:** ^1^ Jiangsu Key Laboratory of Brain Disease and Bioinformation, Research Center for Biochemistry and Molecular Biology Xuzhou Medical University Xuzhou China; ^2^ Department of Genetics, Xuzhou Engineering Research Center of Medical Genetics and Transformation Xuzhou Medical University Xuzhou China; ^3^ Department of Endocrinology Affiliated Hospital of Xuzhou Medical University Xuzhou China; ^4^ The Graduate School Xuzhou Medical University Xuzhou China

**Keywords:** cognitive deficits, MEKK3‐MKK3‐p38 signaling module assembly, p38MAPK/NF‐κB pathway, receptor for advanced glycation end products

## Abstract

In this study, we explored the precise mechanisms underlying the receptor for advanced glycation end products (RAGE)‐mediated neuronal loss and behavioral dysfunction induced by hyperglycemia. We used immunoprecipitation (IP) and GST pull‐down assays to assess the interaction between RAGE and mitogen‐activated protein kinase kinase 3 (MKK3). Then, we investigated the effect of specific mutation of RAGE on plasticity at hippocampal synapses and behavioral deficits in db/db mice through electrophysiological recordings, morphological assays, and behavioral tests. We discovered that RAGE binds MKK3 and that this binding is required for assembly of the MEKK3‐MKK3‐p38 signaling module. Mechanistically, we found that activation of p38 mitogen‐activated protein kinase (MAPK)/NF‐κB signaling depends on mediation of the RAGE‐MKK3 interaction by C‐terminal RAGE (ctRAGE) amino acids (AAs) 2‐5. We found that ctRAGE R2A‐K3A‐R4A‐Q5A mutation suppressed neuronal damage, improved synaptic plasticity, and alleviated behavioral deficits in diabetic mice by disrupting the RAGE‐MKK3 conjugation. High glucose induces direct binding of RAGE and MKK3 via ctRAGE AAs 2‐5, which leads to assembly of the MEKK3‐MKK3‐p38 signaling module and subsequent activation of the p38MAPK/NF‐κB pathway, and ultimately results in diabetic encephalopathy (DE).

## Graphical abstract

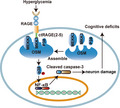

## 1 INTRODUCTION

Diabetes mellitus is a common chronic metabolic disorder that endangers people's health worldwide and is associated with hyperglycemia induced by deficits in insulin secretion or sensitivity (Hannon et al., 2018). Diabetic encephalopathy (DE), which is characterized by cognitive deficits, is one of the most severe complications in patients with diabetes mellitus (Liu et al., 2018; Raffield et al., 2016). The prominent pathological features of DE include atrophy of the brain, impairment of synaptic plasticity, a reduction in the numbers of neurons, and neuroinflammation (Chen et al., 2018; Hu et al., 2020). Prolonged exposure to hyperglycemia leads to a series of structural and functional impairments in brain regions such as the hippocampus, which is essential for cognition and emotion and which exhibits early vulnerability during the progression of DE (Liu et al., 2020; Srikanth et al., 2020).

Studies indicate that the receptor for advanced glycation end products (RAGE) plays a key role in the development of DE (Kim & Song, 2020; Litwinoff et al., 2015). RAGE is a multiple‐ligand receptor that is composed of three extracellular immunoglobulin domains, a transmembrane helix, and a short 42‐amino acid cytoplasmic domain (Litwinoff et al., 2015). Studies have shown that RAGE is associated with the pathogenesis of diabetic complications of the nervous system (Byun et al., 2017; Litwinoff et al., 2015). Over‐accumulation of RAGE in the hippocampus contributes to neuroinflammation and oxidative stress (Han et al., 2019). In addition, RAGE can directly activate the p38MAPK/NF‐κB pathways in the hippocampus, which may underlie synaptic impairments, cognitive deficits, and behavioral changes (Wang et al., 2019). The cytoplasmic domain of RAGE is critical for RAGE‐dependent downstream MAPK signal activation, which ultimately leads to cellular damage (Jules et al., 2013). Inhibition or knockout of RAGE in the brain is accompanied by a reduction in neuronal damage and synaptic dysfunction, and an improvement in behavioral deficits in a mouse model of diabetes and neurodegenerative disease (Momeni et al., 2021b; Toth et al., 2007; Wang et al., 2018) indicated that RAGE may be a potential therapeutic target for DE. Nevertheless, the precise molecular mechanism by which RAGE regulates the p38MAPK pathway remains elusive.

p38MAPK signaling is mediated by scaffold proteins that bind and organize multiple components of the cascade, thereby mediating both the activation of the signal and its localization within a cell (Johnson et al., 2005). The MEKK3‐MKK3‐p38 signaling module is essential for regulation of p38MAPK activation via binding of the osmosensing scaffold protein for MEKK3 (OSM; Hilder et al., 2007). However, it is still not clear whether and/or how RAGE‐dependent p38MAPK/NF‐κB signaling pathway activation is involved in promoting the assembly of the signaling module. In this study, we provide direct evidence that the MEKK3‐MKK3‐p38 cascade is associated with RAGE upregulation of the p38MAPK/NF‐κB pathway via the conjugation of RAGE and MKK3. Importantly, we identify the key structural basis by which RAGE combines directly with MKK3.

## 2 RESULTS

### 2.1 RAGE co‐precipitates with the MEKK3‐MKK3‐p38 signaling module and promotes its assembly

Previous studies have indicated that over‐expression of RAGE promotes p38MAPK/NF‐κB pathway activation, eventually leading to cell damage under high‐glucose conditions (Subedi et al., 2020; Wang et al., 2018). Furthermore, the assembly of the MEKK3‐MKK3‐p38 signaling module is a crucial molecular mechanism underlying p38MAPK signaling (Hilder et al., 2007). In our initial experiments, we sought to investigate the effects of high‐glucose conditions on this pathway in vitro. The conditions of normal (25 mM) and high (80 mM) glucose used in vitro were selected to reflect the conditions in control and diabetic mice. However, owing to the inherent differences between in vitro and in vivo experiments, the glucose levels are not directly comparable (Liu et al., 2016; Meng et al., 2014; Surbala et al., 2020). We compared the levels of RAGE after subjecting cells to high‐glucose conditions for 12, 24 h, or 48 h and found that RAGE was increased significantly after 24 and 48 h (Figure S1a1 and a2); thus, we used 24 h of high‐glucose conditions in the rest of the experiments. High‐glucose conditions enhanced the levels of advanced glycation end products (AGEs) and RAGE, and the specific RAGE antagonist FPS‐ZM1 attenuated the high‐glucose‐induced increases in AGEs and RAGE (Figure S1b, c1 and c2). The level of soluble RAGE (sRAGE) was not significantly different across groups (Figure S1d).

**FIGURE 1:**
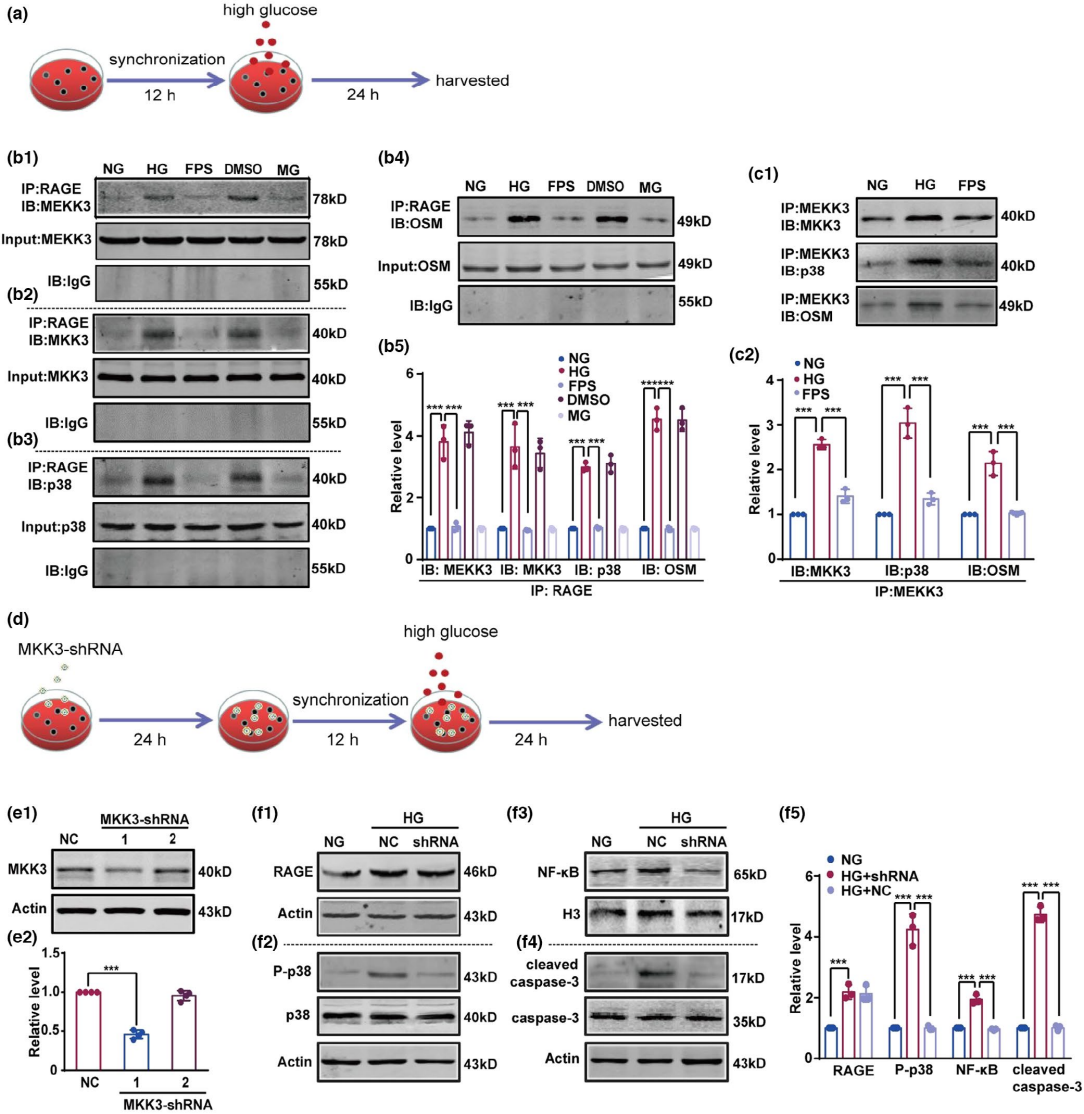
RAGE co‐precipitates with the MEKK3‐MKK3‐p38 signaling module and promotes its assembly under high‐glucose conditions. (a) Schematic of the cell experimental design. (b1–b4) The combination of RAGE and MEKK3‐MKK3‐p38 signaling module was detected with immunoprecipitation followed by immunoblotting with anti‐MEKK3, MKK3, p38, and OSM antibodies, respectively. (b5) The intensity of MEKK3, MKK3, p38, and OSM proteins was represented as fold change to NG group, respectively. Data were calculated by one‐way ANOVA followed by Tukey's test. *F* (4, 10) = 85.42 (MEKK3), 39.55 (MKK3), 167.50 (p38) and 212.10 (OSM), respectively. ***p < 0.001, *n* = 3 in each group. (c1) The assembly of MEKK3‐MKK3‐p38 signaling module was detected with immunoprecipitation followed by immunoblotting with the anti‐MKK3, p38, and OSM antibodies, respectively. (c2) The intensity of the proteins was measured by optical density and presented as fold change with respect to the NG group, respectively. Data were analyzed with a one‐way ANOVA followed by Tukey's test. *F* (2, 6) = 158.80 (MKK3), 84.24 (p38), and 49.89 (OSM), respectively. ***p < 0.001. *n* = 3 in each group. (d) The experimental design was presented. (e1) Representative blots showed MKK3 knockdown. (e2) Relative intensity was assessed using optical density and presented as fold change to NC group. One‐way ANOVA and Tukey's test. *F* (2, 9) = 159.60. ***p < 0.001. *n* = 4 in each group. (f1–f4) The expression of RAGE, P‐p38, and cleaved caspase‐3, and the level of NF‐κB were tested by immunoblotting with the anti‐RAGE, P‐p38, cleaved caspase‐3, and NF‐κB antibodies, respectively. (f5) Relative intensity was displayed as fold change relative to NG group. Data were analyzed by one‐way ANOVA and Tukey's test. *F* (2, 6) = 32.55 (RAGE), 119.40 (P‐p38), 111.00 (NF‐κB), and 588.00 (cleaved caspase‐3), respectively. ***p < 0.001. *n* = 3 in each group

Our data showed that high glucose induced a clear increase in interaction between RAGE and the MEKK3‐MKK3‐p38 signaling module. FPS‐ZM1 blocked the co‐precipitation of RAGE and the MEKK3‐MKK3‐p38 signaling module (Figure 1a,b1–b5). High‐glucose promoted the MEKK3‐MKK3‐p38 cascade and FPS‐ZM1 reversed the interactions between MEKK3 and MKK3, p38, and OSM (Figure 1c1,c2). Furthermore, the interaction between RAGE and the MEKK3‐MKK3‐p38 signaling module caused activation of the p38MAPK/NF‐κB signaling pathway. FPS‐ZM1 specifically decreased the expression of phosphorylated p38 (P‐p38) and cleaved caspase‐3, as well as p65 phosphorylation and nuclear translocation (Figure S1e1–e4, f1 and f2). As shown in Figure 1d, we transfected MKK3‐shRNA into HEK‐293T cells (Figure 1e1,e2) and then measured the levels of RAGE, P‐p38, NF‐κB, and cleaved caspase‐3 under high‐glucose conditions (Figure 1f1–f5). Despite the high‐glucose‐induced increase in RAGE expression, MKK3 knockdown was sufficient to reduce the levels of P‐p38, NF‐κB, and cleaved caspase‐3 (Figure 1f1–f5). High glucose increased cellular apoptosis but inhibition of RAGE diminished this cellular damage (Figure S1g1 and g2). Thus, RAGE promotes the p38MAPK/NF‐κB pathway by facilitating assembly of the MEKK3‐MKK3‐p38 signaling module in high‐glucose conditions.

### 2.2 Interaction between RAGE and the MEKK3‐MKK3‐p38 signaling module accelerates p38MAPK/NF‐κB pathway activation in the hippocampus of diabetic mice

**FIGURE 2:**
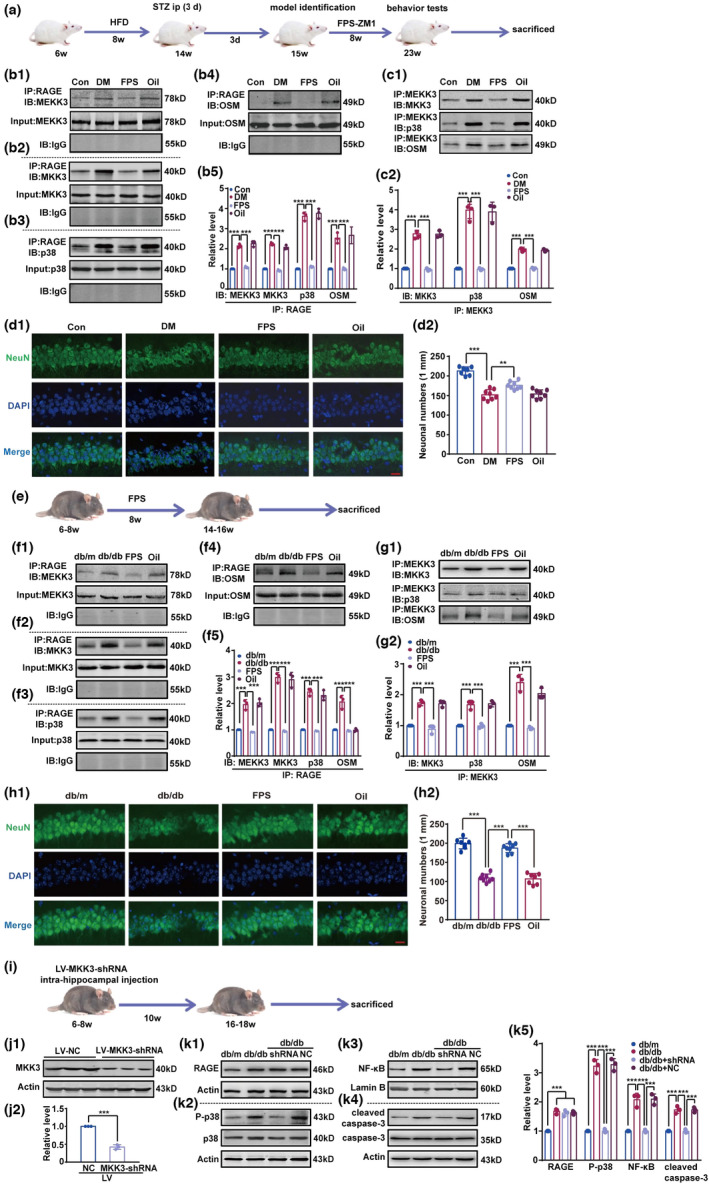
RAGE co‐precipitates with the MEKK3‐MKK3‐p38 signaling module and facilitates p38MAPK/NF‐κB signal activation in diabetic mice hippocampus. (a) Schematic of the experimental design. (b1–b4) The interaction of RAGE and MEKK3‐MKK3‐p38 signaling module in hippocampus was detected with immunoprecipitation followed by immunoblotting with the anti‐MEKK3, MKK3, p38, and OSM antibodies, respectively. B5. The intensity of proteins was represented as fold change to Con group. One‐way ANOVA followed by Tukey's test was used. *F* (3, 8) = 162.40 (MEKK3), 332.20 (MKK3), 265.30 (p38), and 41.33 (OSM), respectively. ***p < 0.001. *n* = 3 in each group. (c1) The assembly of MEKK3‐MKK3‐p38 signaling module in hippocampus was detected with immunoprecipitation followed by immunoblotting with the anti‐MKK3, p38, and OSM antibodies, respectively. (c2) The intensity of the proteins was measured by optical density and displayed as fold change to Con group. Data were analyzed using one‐way ANOVA followed by Tukey's test. *F* (3, 8) = 185.80 (MKK3), 78.02 (p38), and 238.30 (OSM), respectively. ***p < 0.001. *n* = 3 in each group. (d1) Surviving neurons in hippocampal CA1 subregion were measured with immunofluorescence staining by the anti‐NeuN. The number of surviving pyramidal neurons per 1 mm length was counted as neuronal numbers; scale bar is 20 μm (magnification ×400). (d2) One‐way ANOVA followed by Tukey's test was used. *F* (3, 27) = 46.94. Con compared with DM, *p* < 0.001. DM compared with FPS, *p* = 0.001. *n* ≥ 7 from 3 mice in each group. (e) Overview schematic of animal experiment. (f1–f4) Interaction of RAGE and MEKK3‐MKK3‐p38 signaling module in hippocampus were evaluated by immunoprecipitation and immunoblotting with the anti‐MEKK3, MKK3, p38, and OSM antibodies, respectively. (f5) The intensity of proteins was displayed as fold change to db/m group. One‐way ANOVA followed by Tukey's test was used. *F* (3, 8) = 78.45 (MEKK3), 138.80 (MKK3), 137.70 (p38), and 52.13 (OSM), respectively. ***p < 0.001. *n* = 3 in each group. (g1) The assembly of MEKK3‐MKK3‐p38 signaling module in hippocampus was tested by immunoprecipitation and immunoblotting with the anti‐MKK3, p38, and OSM antibodies, respectively. (g2) The intensity of the proteins presented as fold change to the db/m group. Data were analyzed by one‐way ANOVA followed by Tukey's test. *F* (3, 8) = 60.10 (MKK3), 53.60 (p38), and 64.17 (OSM), respectively. ***p < 0.001. *n* = 3 in each group. (h1) Surviving neurons in hippocampal CA1 subregion were measured by immunofluorescence staining with the anti‐NeuN. The number of surviving pyramidal neurons per 1‐mm length was counted. Scale bar is 20 μm (magnification ×400). (h2) One‐way ANOVA and Tukey's test were used. *F* (3, 27) = 116.00. ***p < 0.001. *n* ≥ 7 from three mice in each group. (i) Experimental design of MKK3 knockdown was displayed. (j1) Typical blots showed MKK3 knockdown in hippocampus of db/db mice. (j2) Relative intensity was evaluated using optical density and displayed as fold change to NC group. T test was used and *t* = 15.04, *df* = 4. ***p < 0.001. *n* = 3 in each group. Data were shown as mean ± SD and source data were provided as a Source Data file. (k1–k4) The expression of RAGE, P‐p38, and cleaved caspase‐3, and the level of NF‐κB were tested by immunoblotting with the anti‐RAGE, P‐p38, cleaved caspase‐3, and NF‐κB antibodies, respectively. (k5) Relative intensity was displayed as fold change to the NG group. Data were analyzed by one‐way ANOVA and Tukey's test. *F* (3, 8) = 59.71 (RAGE), 211.70 (P‐p38), 54.96 (NF‐κB) and 85.15 (cleaved caspase‐3), respectively. ***p < 0.001. *n* = 3 in each group

Next, we investigated the effect of RAGE on the p38MAPK/NF‐κB pathway in vivo. First, we used mice with streptozotocin (STZ)‐induced diabetes (see Section 4). Diabetic mice had higher level of RAGE than control mice, and both 1 and 2 mg FPS‐ZM1 significantly reduced RAGE expression (Figure S2a1 and a2). We thus selected 1 mg FPS for further experiments. As displayed in Figure 2a, we measured co‐precipitation of RAGE with MEKK3, MKK3, p38, and the scaffold protein OSM using an immunoprecipitation assay (Figure 2b1–b4). Mice with STZ‐induced diabetes had significantly greater levels of co‐precipitation of RAGE with components of the MEKK3‐MKK3‐p38 signaling module, which were abrogated by the RAGE antagonist, FPS‐ZM1 (Figure 2b1–b5). Furthermore, diabetic mice had higher levels of AGEs and RAGE than control mice, but FPS‐ZM1 blocked this augmentation of AGEs and RAGE (Figure S2b, c1 and c2). Data from immunoprecipitation assays show that the assembly of the MEKK3‐MKK3‐p38 signaling module was increased, and that administration of FPS‐ZM1 suppressed this increase (Figure 2c1 and c2).

Hyperglycemia induced p38 phosphorylation, p65 phosphorylation, NF‐κB nuclear translocation, and caspase‐3 activation. Likewise, inhibition of RAGE and the MEKK3‐MKK3‐p38 interaction significantly reduced p38MAPK/NF‐κB pathway activation (Figure S2d1‐d4, and e1 and e2). Diabetic mice had a lower number of surviving neurons in the hippocampal CA1 subregion compared with control mice, but FPS‐ZM1 reduced this neuronal loss (Figure 2d1–d2). Although the weight of diabetic mice was higher than control mice from the 10th week to the 23th week, there were no differences in weight between the untreated diabetic mice and the diabetic mice treated with FPS‐ZM1 or corn oil (Figure S3a). Diabetic mice had higher blood glucose levels, lower glucose tolerance, and greater water consumption than control mice; however, neither FPS‐ZM1 nor corn oil had any effect on blood glucose, glucose tolerance, and water intake in diabetic mice (Figure S3b‐d).

Next, we investigated RAGE and the p38MAPK/NF‐κB pathway in the db/db mice, and the experimental schedule was shown in Figure 2e. The interaction between RAGE and the MEKK3‐MKK3‐p38 signaling module, and the aggregation of the module were all blocked by FPS‐ZM1 (Figure 2f1–f5, and g1 and g2). Similarly, inhibition of RAGE reduced neuronal loss in the hippocampal CA1 subregion of db/db mice (Figure 2h1,h2). As shown in Figure 2i, we microinjected LV‐MKK3‐shRNA into the hippocampal CA1 subregion (Figure 2j1,j2). db/db mice had high RAGE expression, but MKK3 knockdown clearly reduced the levels of P‐p38, NF‐κB, and cleaved caspase‐3 in hippocampus (Figure 2k1–k5). These results indicate that p38MAPK/NF‐κB pathway activation is associated with the interaction between RAGE and the MEKK3‐MKK3‐p38 signaling module.

### 2.3 RAGE combined with the MEKK3‐MKK3‐p38 signaling module accelerates diabetes‐induced behavior deficits

**FIGURE 3:**
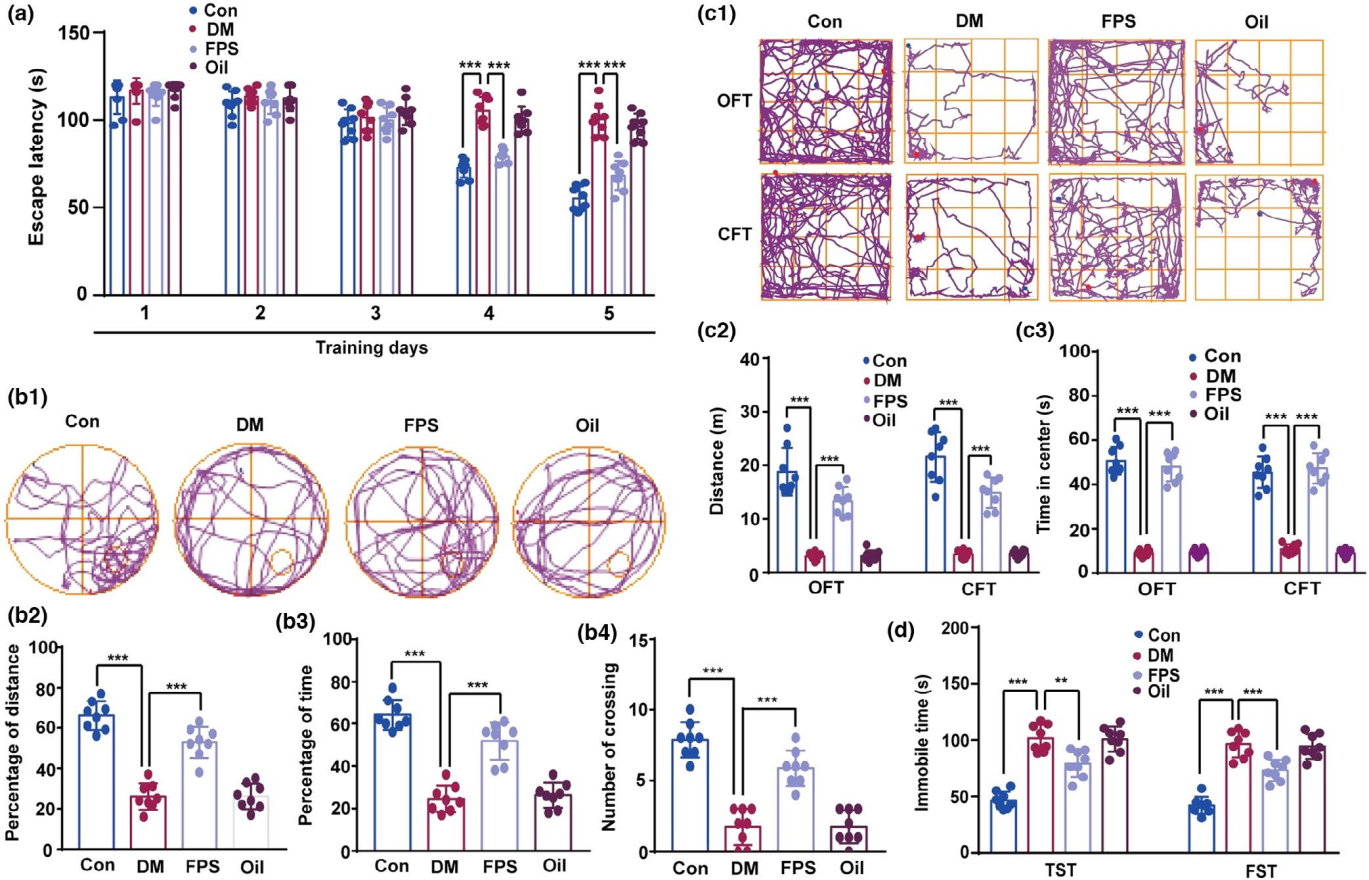
Inhibition of RAGE binding to the MEKK3‐MKK3‐p38 signaling module alleviates diabetic encephalopathy. (a) The average latency for mice to find the hidden platform. Data were analyzed by two‐way ANOVA and repeated measures followed by Tukey's test. On the 4th day, Con compared with DM, and DM compared with FPS, *p* < 0.001. q (8, 40) = 12.89 (Con/DM) and 10.20 (DM/FPS); on the 5th day, q (8, 40) = 17.84 (Con/DM) and 12.75 (DM/FPS). ***p < 0.001. *n* = 8 in each group. (b1) Track maps of the probe trial performed on the 6th day without the platform. (b2–b4) Distance and time spent in target quadrant, and the number of the platform crossings on the 6th day with the platform was taken away were displayed. Data were analyzed by one‐way ANOVA followed by Tukey's test. *F* (3, 28) = 66.81 (B2), 60.46 (B3), and 49.37 (B4), respectively. ***p < 0.001, *n* = 8 in each group. (C1) Track maps of mice in the OFT (upper) and CFT (lower). (c2 and c3) Total distance traveled and the time in the center were analyzed. One‐way ANOVA followed by Tukey's test was used. *F* (3, 28) = 69.78 (OFT) and 84.17 (CFT) for the total distance, respectively. *F* (3, 28) = 199.30 (OFT) and 135.30 (CFT) for the time in center, respectively. ***p < 0.001. *n* = 8 in each group. (d) Immobility times of mice in TST and FST were evaluated. Data were analyzed by one‐way ANOVA followed by Tukey's test. *F* (3, 28) = 45.75 (TST) and 50.51 (FST), respectively. Con compared with DM in TST and FST, *p* < 0.001. DM compared with FPS, *p* = 0.0014 in TST and *p* < 0.001 in FST. ***p < 0.001 and **p < 0.01. *n* = 8 in each group

As shown in Figure 3a, on Days 4 and 5 of the acquisition phase of the Morris water maze (MWM), the escape latency of diabetic mice was significantly longer than that of control mice. Of note, FPS‐ZM1 restored the ability of diabetic mice to find the hidden platform (Figure 3a). During the probe trial, the distance and time spent in the target quadrant and the number of times the mice crossed the previous position of the platform were all reduced in diabetic mice. However, diabetic mice treated with FPS‐ZM1 showed retention of spatial memory in the probe test (Figure 3b1–b4). Diabetic mice had a significant reduction in the total distance traveled and time spent in the center of the open field test (OFT) and closed field test (CFT), relative to controls, but locomotor activity was rescued by FPS‐ZM1 (Figure 3c1–c3). As shown in Figure 3d, diabetic mice had a significant increase in the duration of immobility in both the tail suspension test (TST) and forced swim test (FST), but FPS‐ZM1 alleviated this depressive behavior. These data support the hypothesis that the interaction between RAGE and the MEKK3‐MKK3‐p38 signaling module exacerbates hyperglycemia‐induced cognitive deficits and depression.

### 2.4 RAGE directly binds MKK3 through its RKRQ motif

**FIGURE 4:**
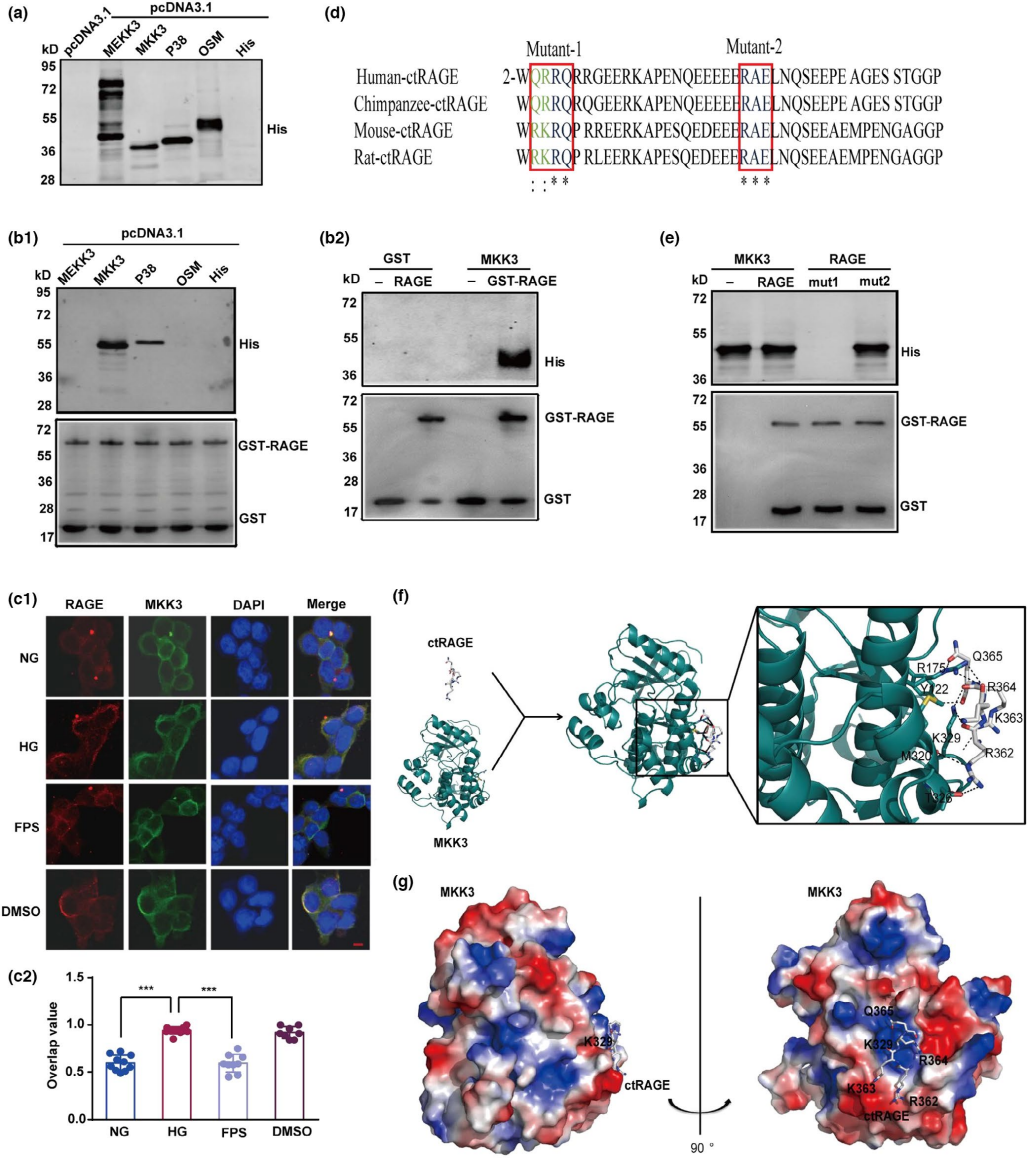
AAs 2‐5 is a key domain of mouse ctRAGE binding to MKK3. (a) His‐pcDNA3.1‐MEKK3, His‐pcDNA3.1‐MKK3, His‐pcDNA3.1‐p38, His‐pcDNA3.1‐OSM, and His‐pcDNA3.1 were over‐expressed in HEK‐293T cells and tested by immunoblotting with anti‐His. (b1) RAGE directly binds MKK3. GST‐tagged RAGE was expressed in BL21 cells and then subjected to GST pull‐down analysis. MKK3 and p38 in the HEK‐293T cell lysate were pulled down by GST‐tagged RAGE. (b2) Typical blots of RAGE binding to MKK3. GST and GST‐tagged RAGE were expressed in BL21 cells and then subjected to GST pull‐down analysis. MKK3 in the HEK‐293T cell lysate was pulled down by GST‐tagged RAGE. (c1 and c2) Typical laser‐scanning confocal microscopy images and overlap values showed co‐localization of RAGE and MKK3 in HEK‐293T cells induced by high glucose. RAGE was marked in red, MKK3 was marked in green, cellular nucleus was labeled in blue by DAPI, and the co‐localization of RAGE and MKK3 was presented in yellow. Scale bar is 10 µm (magnification ×400). Overlap data were analyzed by one‐way ANOVA followed by Tukey's test. *F* (3, 34) = 68.90. ***p < 0.001. *n* ≥ 8 in each group. Data were shown as mean ± SD. (d) Homology of C‐terminal RAGE (ctRAGE) in different species with two mouse mutant domains (mutation 1: R2A/K3A/R4A/Q5A; mutation 2: R23A/E25A) were showed.: indicated highly homologous (green) and * indicated completely homologous (blue). (e) RAGE mutation 1 specifically inhibited RAGE‐MKKE interaction. GST‐tagged RAGE mutation 1 and mutation 2 were expressed in BL21 cells and then subjected to GST pull‐down analysis. MKK3 in the HEK‐293T cell lysate was pulled down by GST‐tagged RAGE mutation 2, and RAGE mutation could not interact with MKK3. (f and g) Cartoon linear docking figure and vacuum electrostatics map showed spatial details of ctRAGE‐MKK3 interaction. RKRQ motif in ctRAGE was shown in sticks (colored by atom), and related residues of MKK3 were also shown as sticks. The polar contacts bond between MKK3 and RAGE is represented by dotted lines. The vacuum electrostatics map showed positive‐charged surface (blue), negative‐charged surface (red), and neutral surface (white) of MKK3. K329 on MKK3 and RKRQ motif were labeled. A Swiss‐model server and protein data bank (PDB) database were used to analyze the spatial structure of MKK3 and ctRAGE. The docking model was generated by Autodock Vina (version 1.1.2). The model figures representing protein interaction were drawn in PyMOL (version 0.99)

It is widely accepted that the intracellular C‐terminal of RAGE (ctRAGE) is the crucial domain for activating downstream signaling (Ishihara et al., 2003). GST pull‐down assay was performed to address the structural basis by which ctRAGE binds the target protein; we generated recombinant GST‐pGEX‐4T‐1‐RAGE, and His‐tagged pcDNA3.1 MEKK3, MKK3, p38, and OSM plasmids. Results showed over‐expression of His‐tagged MEKK3, MKK3, p38, and OSM proteins in HEK‐293T cells (Figure 4a). Data indicated that MKK3, not MEKK3 or OSM, binds GST‐fusion ctRAGE directly, although a relatively weak interaction between RAGE and p38 cannot be ruled out (Figure 4b1). As shown in Figure 4b2, GST‐pGEX‐4T‐1 did not bind His‐tagged pcDNA3.1‐MKK3. After high‐glucose stimulation, co‐localization of RAGE and MKK3 was observed in a large number of HEK‐293T cells. The co‐localization signal was significantly weaker under FPS‐ZM1 treatment (Figure 4c1,c2).

We next focused on identifying the RAGE motif that is critical for binding to MKK3. On the basis of the reported domains in RAGE (Manigrasso et al., 2016; Zhou et al., 2018), we generated two recombinant mouse ctRAGE mutations (R2A‐K3A‐R4A‐Q5A and R23A‐E25A; Figure 4d). GST pull‐down data indicate that ctRAGE with a mutation in amino acids (AAs) 2‐5, but not AAs 23‐25, blocked RAGE and MKK3 conjugation (Figure 4e). Thus, the AAs 2‐5 motif in ctRAGE is crucial for the RAGE‐MKK3 interaction. ctRAGE with a mutation in AAs 2‐5 was selected for the subsequent experiments. We next performed molecular docking to discover the spatial features of the ctRAGE‐MKK3 interaction. Of note, docking results showed that Y122, R175, M320, T326, and K329 in MKK3 are critical in the formation of the ctRAGE‐MKK3 complex as they are involved in the polar contacts between ctRAGE and MKK3 (Figure 4f). Of these, R175 and K329 were identified as the “central” residues since they interact simultaneously with multiple residues in the RKRQ motif. The vacuum electrostatic map of MKK3 showed that K329, together with R175 and other nearby residues with positively charged side chain, forms a positively charged “semi‐pocket” that, to a large extent, fits the spatial structure of RKRQ motif (Figure 4g). These results indicate that MKK3 is the target protein through which RAGE interacts with the MEKK3‐MKK3‐p38 signaling module, and that ctRAGE AAs 2‐5 is the key RAGE motif underlying binding to MKK3.

### 2.5 R2A‐K3A‐R4A‐Q5A mutation of ctRAGE inhibits co‐precipitation and co‐localization with MKK3 in HT22 cells

**FIGURE 5:**
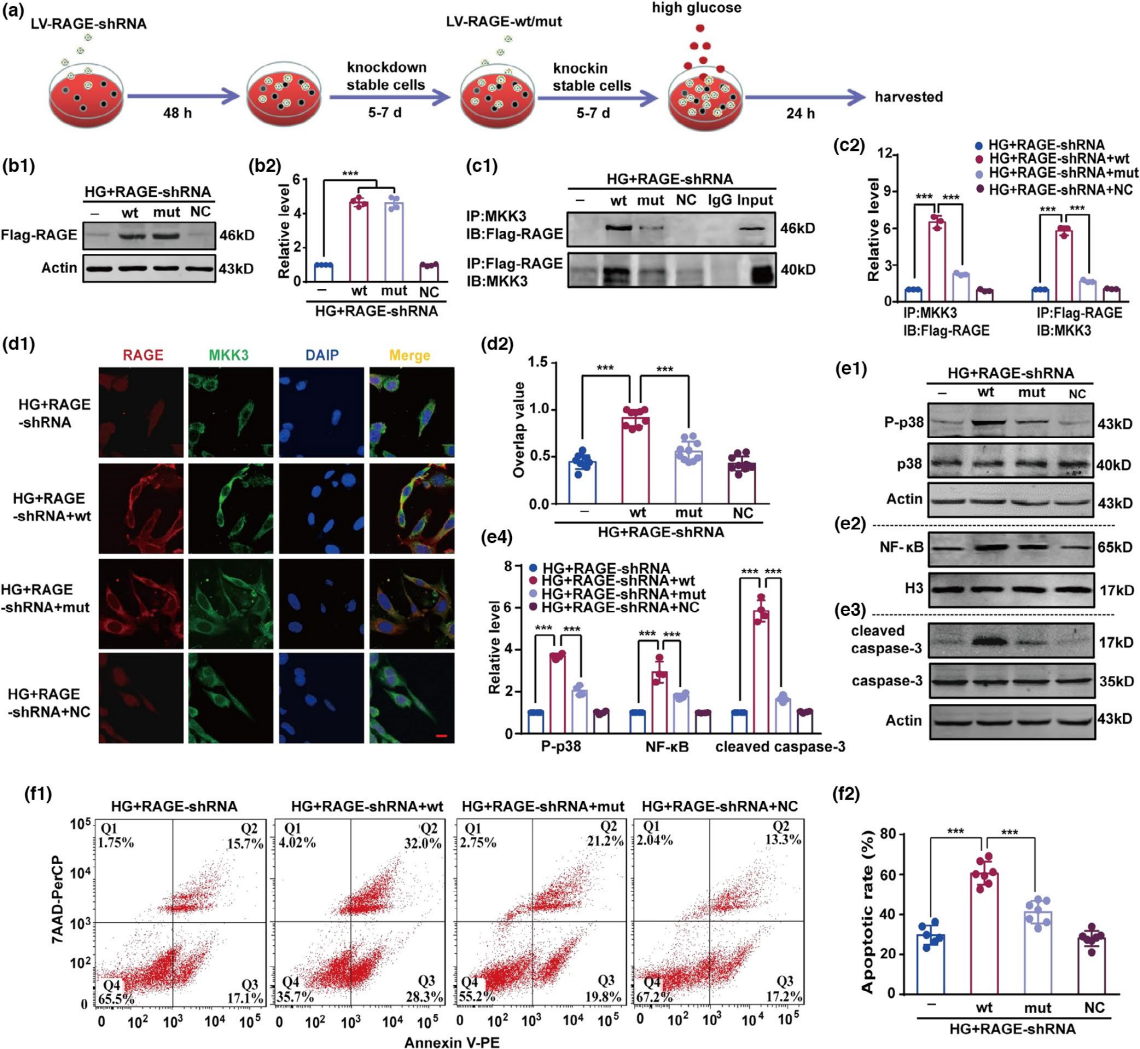
In a high‐glucose environment, RAGE mutation decreases p38 phosphorylation, NF‐κB nuclear transcription, and caspase‐3 activation in HT22 cells by interrupting the RAGE‐MKK3 interaction. (a) Schematic of RAGE inhibition and over‐expression in HT22 cells. (b1) The expression of Flag‐RAGE in high glucose condition was detected by immunoblotting with the anti‐Flag antibody. (b2) Optical density was emerged as fold change to HG + RAGE‐shRNA group. Results were analyzed using one‐way ANOVA followed by Tukey's test. *F* (3, 12) = 378.80. ***p < 0.001. *n* = 4 in each group. (c1) The interaction of Flag‐RAGE and MKK3 was detected with immunoprecipitation followed by immunoblotting with the anti‐Flag and MKK3 antibodies, respectively. (c2) Optical density was shown as fold change to HG + RAGE‐shRNA group. Relative levels were analyzed using one‐way ANOVA followed by Tukey's test. *F* (3, 8) = 321.10 (Flag‐RAGE) and 441.50 (MKK3), respectively. ***p < 0.001. *n* = 3 in each group. (d1) RAGE mutation blocked co‐localization of RAGE and MKK3 after high glucose stimulation. Wild‐type and mutated RAGE were transfected into HT22 cells, and Immunofluorescence was used to detect the co‐localization of RAGE and MKK3. Flag‐RAGE was marked in red, MKK3 was marked in green, cellular nuclei were labeled in blue by DAPI, and the co‐localization of RAGE and MKK3 was displayed in yellow. Scale bar is 10 µm (magnification ×400). (d2) The overlap value was calculated by one‐way ANOVA followed by Tukey's test. *F* (3, 34) = 62.72. ***p < 0.001. *n* ≥ 8 in each group. (e1–e3) The expression of P‐p38 and cleaved caspase‐3, and the level of NF‐κB in high‐glucose conditions were detected by immunoblotting with the anti‐P‐p38, cleaved caspase‐3, and NF‐κB antibodies, respectively. (e4) Relative intensity was presented as fold change to HG + RAGE‐shRNA group. Results were calculated by one‐way ANOVA followed by Tukey's test. *F* (3, 12) = 344.90 (p38), 49.54 (NF‐κB), and 297.20 (cleaved caspase‐3), respectively. ***p < 0.001. *n* = 3 in each group. (f1) Representative cellular flow cytometry images emerged cellular apoptosis. Dead cells in Q1, late apoptotic cells in Q2, viable apoptotic cells in Q3, and normal cells in Q4. (f2) Apoptotic rate (number of cells in Q2 and Q3/total number of cells) was presented. One‐way ANOVA followed by Tukey's test was used. *F* (3, 23) = 59.68. ***p < 0.001. *n* ≥ 6 in each group

We next investigated whether mutant RAGE could directly block RAGE‐MKK3 interaction and p38MAPK/NF‐κB pathway activation; the experimental schedule was presented in Figure 5a. To eliminate interference from endogenous RAGE, we first transfected RAGE‐shRNA into HT22 cells (Figure S4a1). As shown in Figure S4a2 and a3, shRNA1 and shRNA2 significantly reduced endogenous RAGE in HT22 cells. We selected shRNA2 in lentivirus (LV) with a multiplicity of infection (MOI) of 10 for use in further experiments. Subsequently, Flag‐tagged LV with either wild‐type ctRAGE or R2A‐K3A‐R4A‐Q5A mutated ctRAGE was transfected into RAGE‐knockdown HT22 cells (Figure S4b1 and b2). We then assessed the effect of high‐glucose on the expression of wild‐type or mutated ctRAGE in RAGE‐knockdown HT22 cells, and found that both were increased (Figure 5b1,b2). Mutant ctRAGE attenuated RAGE‐MKK3 conjugation in high‐glucose conditions (Figure 5c1,c2). MKK3 had significantly lower co‐localization with mutant ctRAGE (AAs 2‐5) than with wild‐type ctRAGE (Figure 5d1,d2).

We detected p38MAPK/NF‐κB pathway activation by immunoblotting (Figure 5e1–e3). Although the levels of p38MAPK/NF‐κB were activated with wild‐type ctRAGE treatment, ctRAGE with R2A‐K3A‐R4A‐Q5A mutation restrained such increases (Figure 5e1–e4). Furthermore, mutation of AAs 2‐5 in ctRAGE protected HT22 cells from the effect of high‐glucose, whereas wild‐type ctRAGE had no such protective effect (Figure 5f1,f2). Taken together, these results suggest that high‐glucose‐induced p38MAPK/NF‐κB pathway activation and cell injury are alleviated by mutation of AAs 2‐5 in ctRAGE, which blocks the interaction between RAGE and MKK3.

### 2.6 AAs 2‐5 mutation of ctRAGE decreases p38MAPK/NF‐κB signaling pathway activation through inhibition of the RAGE‐MKK3 interaction in hippocampus of db/db mice

**FIGURE 6:**
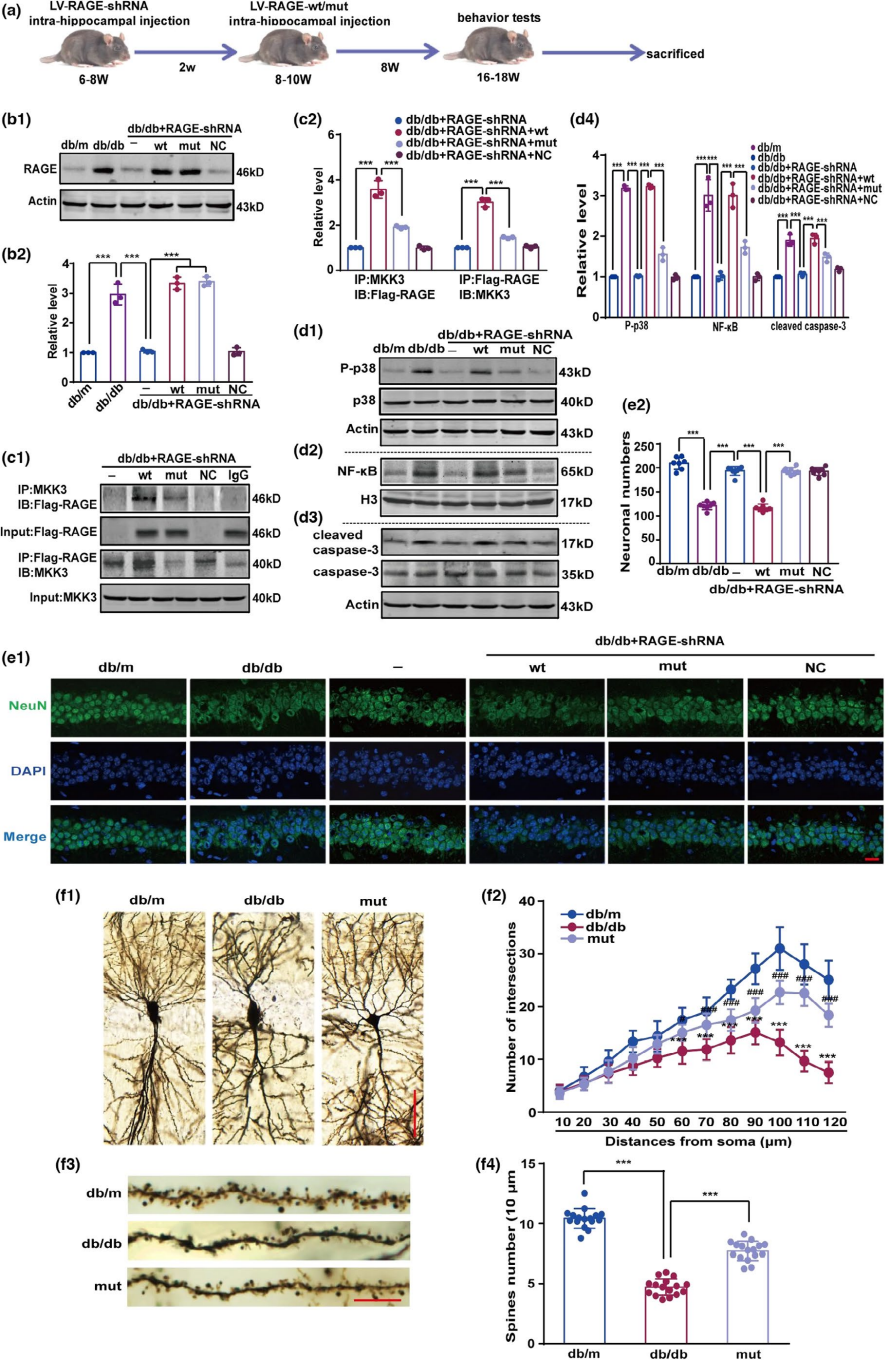
RAGE mutation decreases p38 phosphorylation, NF‐κB nuclear transcription, and activation of caspase‐3 in hippocampus of db/db mice by blocking co‐precipitation of RAGE and MKK. (a) Schematic overview of the experimental design. (b1) The expression of Flag‐RAGE in hippocampus of db/db mice was detected by immunoblotting with the RAGE antibody. (b2) The intensity was represented as fold change to db/m group. Data were analyzed by one‐way ANOVA followed by Tukey's test. *F* (5, 12) = 127.70. ***p < 0.001. *n* = 3 in each group. (c1) The interaction of Flag‐RAGE and MKK3 was detected with immunoprecipitation followed by immunoblotting with the anti‐Flag and MKK3 antibodies, respectively. (c2) Optical density was displayed as fold change to db/db + RAGE‐shRNA group. Results were calculated by one‐way ANOVA followed by Tukey's test. *F* (3, 8) = 106.10 (Flag‐RAGE) and 212.10 (MKK3), respectively. ***p < 0.001. *n* = 3 in each group. (d1–d3) Expression of P‐p38 and cleaved caspase‐3, and the level of NF‐κB were detected by immunoblotting with the anti‐P‐p38, cleaved caspase‐3 NF‐κB antibodies. (d4) Relative intensity of protein was presented as fold change to db/m group. One‐way ANOVA followed by Tukey's test. *F* (5, 12) = 581.20 (P‐p38), 62.14 (NF‐κB) and 58.09 (cleaved caspase‐3), respectively. ***p < 0.01. *n* = 3 in each group. (e1) Surviving neurons in the hippocampal CA1 subregion were assessed with immunofluorescence staining by the anti‐NeuN, and the number of neurons was evaluated as the numbers of surviving pyramidal neurons per 1‐mm length. Scale bar is 20 μm (magnification ×400). (e2) Overlap value was calculated. One‐way ANOVA followed by Tukey's test. *F* (5, 39) = 154.50. ***p < 0.001. *n* ≥ 7 from 3 mice in each group. (f1) Branching morphologically of neurons in hippocampal CA1 subregion was shown by Golgi staining. Scale bar is 50 µm (magnification ×200) (f2) Dendritic intersection number was analyzed by Sholl analysis. Two‐way repeated measures ANOVA followed by Tukey's test. In 60 μm, db/m compared with db/db, *p* < 0.001; db/db compared with mut (db/db + RAGE‐shRNA + mut group), *p* = 0.025. q (12, 396) = 9.07 (dbm/db/db) and 5.36 (db/db/mut). Distances from 70 μm to 120 μm, db/m compared with db/db and db/db compared with mut, *p* < 0.001. In 70 μm, q (12, 396) = 10.98 (dbm/db/db) and 7.15 (db/db/mut); in 80 μm, q (12, 396) = 14.18 (dbm/db/db) and 5.87 (db/db/mut); in 90 μm, q (12, 396) = 18.52 (dbm/db/db) and 6.39 (db/db/mut); in 100 μm, q (12, 396) = 27.33 (dbm/db/db) and 14.56 (db/db/mut); in 110 μm, q (12, 396) = 28.09 (dbm/db/db) and 19.66 (db/db/mut); in 120 μm, q (12, 396) = 26.94 (dbm/db/db) and 16.60 (db/db/mut). ***p < 0.001, ^#^ *p* < 0.05, ^###^ *p* < 0.001. *n* = 12 from 4 mice (3 sections per mouse). (f3) Dendritic spine density and morphology for each group were present. Scale bar is 10 µm (magnification ×600). (f4) Dendritic spine number was calculated with Image J, and data were analyzed by one‐way ANOVA followed with Tukey's test. *F* (2, 45) = 219.80. ***p < 0.001. *n* = 16 from four mice (four sections per mouse)

The adverse effects of diabetes on the brain are especially pronounced in the hippocampus, which is highly susceptible to hyperglycemia (Satrom et al., 2018). Diabetes‐induced injury to hippocampal neurons likely involves in p38 and NF‐κB activation (Han et al., 2019; Liu et al., 2019). Thus, we asked whether mutated RAGE could affect the p38MAPK/NF‐κB pathway in hippocampus, and the experimental schedule was shown in Figure 6a. To avoid interference by endogenous RAGE, LV‐RAGE‐shRNA was microinjected bilaterally into the hippocampal CA1 regions. The needle passage was marked with bromophenol blue to ensure the injection site was accurate (Figure S5a). Application of either 1 μl or 2 μl LV‐RAGE‐shRNA decreased RAGE expression in the hippocampus (Figure S5b1 and b2) and 1 μl LV‐RAGE‐shRNA was used in the subsequent studies. Wild‐type or R2A‐K3A‐R4A‐Q5A‐mutated ctRAGE was injected bilaterally into the hippocampus 2 weeks after injection of LV‐RAGE‐shRNA. Immunoblot analysis (Figure 6b1) showed unambiguously that the level of RAGE in the db/db + RAGE‐shRNA + wt group was similar to the levels in the db/db + RAGE‐shRNA + mut group (Figure 6b1,b2). Meanwhile, GFP‐tagged LV‐RAGE‐wt and LV‐RAGE‐mut were over‐expressed in hippocampal sections, while endogenous RAGE was inhibited by LV‐RAGE‐shRNA (Figure S5c). Immunoprecipitation data (Figure 6c1) show that mutated ctRAGE dramatically reduced the binding between RAGE and MKK3, whereas wild‐type ctRAGE bound well to MKK3 (Figure 6c1,c2). We also found that expression of the p38MAPK/NF‐κB pathway was higher in db/db mice than in db/m mice (Figure 6d1–d4). Of note, p38MAPK/NF‐κB pathway activation was completely inhibited by R2A‐K3A‐R4A‐Q5A mutation (Figure 6d1–d4).

As indicated in Figure 6e1–e2, diabetes caused neuronal loss in the hippocampal CA1 subfield. Compared with wild‐type ctRAGE, mutated ctRAGE significantly diminished the loss of pyramidal neurons in the hippocampal CA1 subfield (Figure 6e1,e2). Golgi staining showed that the degree of interlacing of pyramidal neurons in hippocampal CA1 subregion was significantly lower in db/db mice than in db/m mice, and ctRAGE mutation increased the degree of dendritic overlap (Figure 6f1,f2). Persistent hyperglycemia reduced sporadic network morphology, lessened the degree of divergence, and reduced the number of pyramidal‐neuron dendrites, but mutated ctRAGE rescued the anomalous morphology and dendritic spine count (Figure 6f3,f4).

Furthermore, although weight change in db/db mice was higher than in db/m mice, we did not detect any significant differences in mice receiving wild‐type ctRAGE or mutated ctRAGE (Figure S6a). db/db mice had higher blood glucose levels, worse glucose tolerance, and greater water intake than db/m mice; however, neither RAGE knockdown nor administration of wild‐type ctRAGE or mutated ctRAGE had any effect on these basic symptoms of diabetes (Figure S6b–d). Taken together, these data indicate that, in db/db mice, mutation of ctRAGE (R2A‐K3A‐R4A‐Q5A) disrupts its ability to bind MKK3, which in turn reduces activation of the p38MAPK/NF‐κB signaling pathway and decreases damage to hippocampal neurons.

### 2.7 AAs 2‐5 mutation of ctRAGE improves synaptic function in db/db mice

**FIGURE 7:**
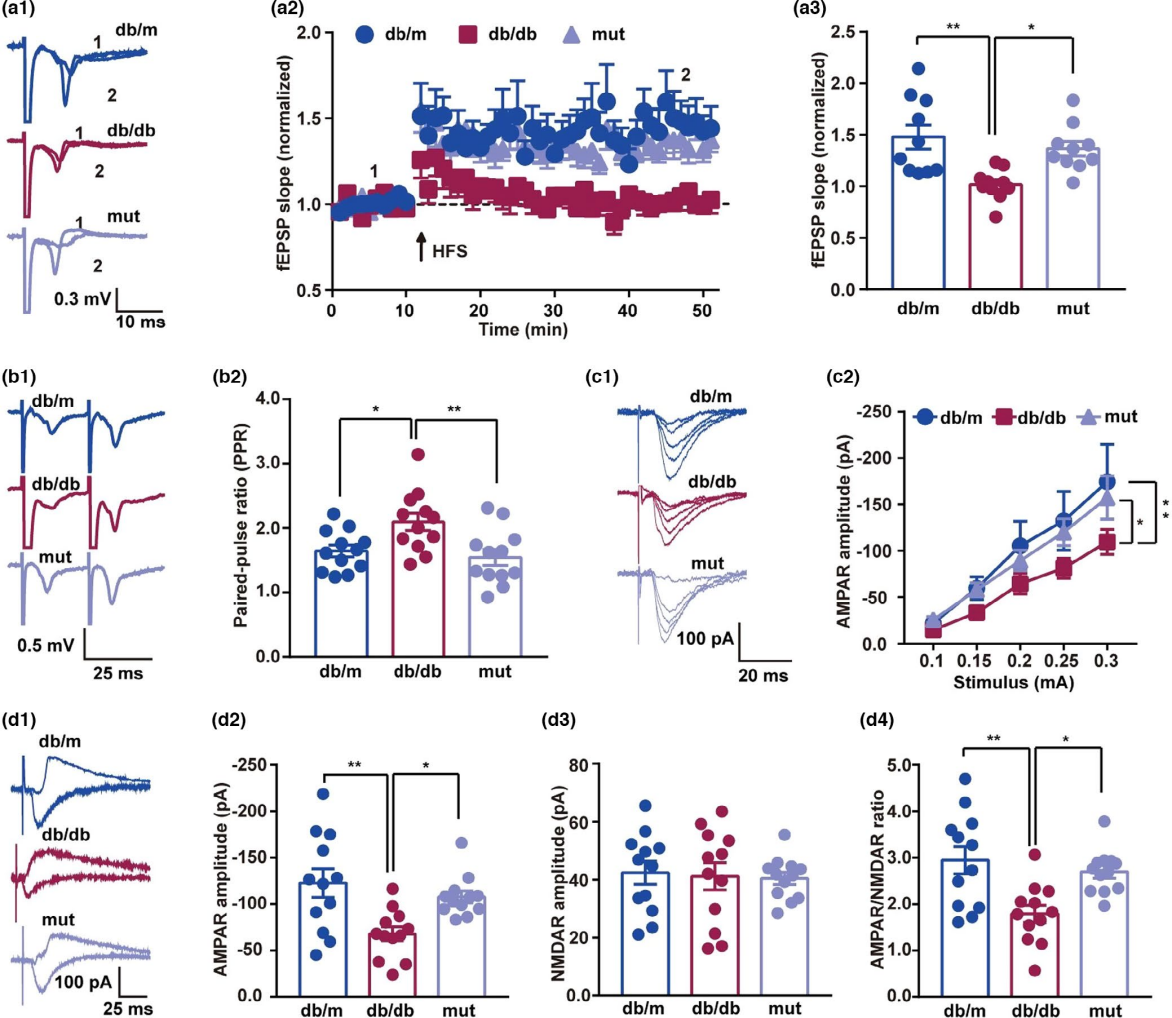
RAGE mutation ameliorates impairments in synaptic plasticity in db/db mice. (a1 and a2) Representative traces and time course of fEPSP slopes in hippocampal CA1 subregion from three groups. (a3) RAGE mutation rescued the LTP fEPSP deficit in db/db mice. The relative increase in fEPSP during the last 10 min of recording was analyzed with one‐way ANOVA followed by Tukey's test. *F* (2, 27) = 8.18. db/m compared with db/db, *p* = 0.0017. db/db compared with mut (db/db + RAGE‐shRAN + mut group), *p* = 0.0182. *n* = 10 from 5 mice in each group (two slices per mouse). (b1) Representative PPRs calculated from fEPSPs with a 25‐ms interval between pulses. (b2) RAGE mutation rescued the PPR increments seen in db/db mice. Data were analyzed with one‐way ANOVA followed by Tukey's test. *F* (2, 33) = 6.13. db/m compared with db/db, *p* = 0.0291. db/db compared with mut, *p* = 0.0066. *n* = 12 from 4 mice in each group (three slices per mouse). (c1) Representative input–output curves for AMPAR EPSCs recorded in whole‐cell patch‐clamping mode. (c2) RAGE mutation rescued the decrements in AMPAR EPSCs seen in db/db mice. Data were analyzed by two‐way ANOVA followed with Tukey's test. *F* (4, 165) = 22.79. db/m compared with db/db, *p* = 0.0030. db/db compared with mut, *p* = 0.0290. *n* = 12 from 4 mice in each group (three slices per mouse). (d1) Representative traces of AMPAR, NMDAR EPSCs, and their ratios recorded in whole‐cell patch‐clamping mode. The stimulating intensity was 0.2 mA. (d2) RAGE mutation rescued the decrements in AMPAR EPSCs seen in db/db mice. Data were analyzed by one‐way ANOVA followed with Tukey's test. *F* (2, 33) = 7.27. db/m compared with db/db, *p* = 0.0023. db/db compared with mut, *p* = 0.0296. *n* = 12 from 4 mice in each group (three slices per mouse). (d3) No change in ionotropic NMDAR EPSCs in the three groups. Data were analyzed by one‐way ANOVA followed by Tukey's test. *F* (2, 33) = 0.07. db/m compared with db/db, *p* = 0.9731. db/db compared with mut, *p* = 0.9900. *n* = 12 from 4 mice in each group (three slices per mouse). (d4) RAGE mutation rescued the decrements in the AMPAR/NMDAR ratios seen in db/db mice. Data were analyzed by one‐way ANOVA followed by Tukey's test. *F* (2, 33) = 7.88. db/m compared with db/db, *p* = 0.0018. db/db compared with mut, *p* = 0.0154. *n* = 12 from four mice in each group (three slices per mice)

RAGE signaling is required for synaptic dysfunction in diabetes and neurodegenerative disease (Momeni et al., 2021a; Zhang et al., 2014). We therefore examined the effect of specific mutation of RAGE on the maintenance of long‐term potentiation (LTP), a form of synaptic plasticity that is widely thought to be the basis of learning and memory in db/db mice. Electrophysiological recordings in hippocampal slices showed that high‐frequency stimulation (HFS)‐induced LTP of field excitatory postsynaptic potentials (fEPSPs) was significantly inhibited in db/db mice, but not in db/db mice injected with mutated ctRAGE (Figure 7a1–a3). To determine whether there were presynaptic changes in the hippocampus of these mice, we measured the paired‐pulse ratio (PPR) of the fEPSPs. The PPR showed a significant increase with 25 ms interval in db/db mice, but ctRAGE mutation rescued this effect (Figure 7b1,b2).

To address whether changes in synaptic plasticity were due to impaired α‐amino‐3‐hydroxy‐5‐methyl‐4‐isoxazole‐propionic acid receptor (AMPAR)/N‐methyl‐D‐aspartic acid receptor (NMDAR) function, we recorded AMPAR and NMDAR‐mediated excitatory postsynaptic currents (EPSCs) in whole‐cell patch‐clamping mode (Figure 7c1,d1). The input–output curves of AMPAR EPSCs were decreased in db/db mice; but mutation of AAs 2‐5 in ctRAGE markedly improved AMPAR EPSCs in db/db mice (Figure 7c2,d2). However, NMDAR EPSCs were unchanged in all groups (Figure 7d3). Figure 7d4 showed the AMPAR/NMDAR ratio was lower in db/db mice than in db/m mice, but mutant RAGE increased this ratio (Figure 7d4). Taken together, these findings demonstrate that ctRAGE mutation alleviates impairments in synaptic plasticity by modulating AMPAR function.

### 2.8 ctRAGE AAs 2‐5 mutation ameliorates behavioral deficits associated with diabetic encephalopathy

**FIGURE 8:**
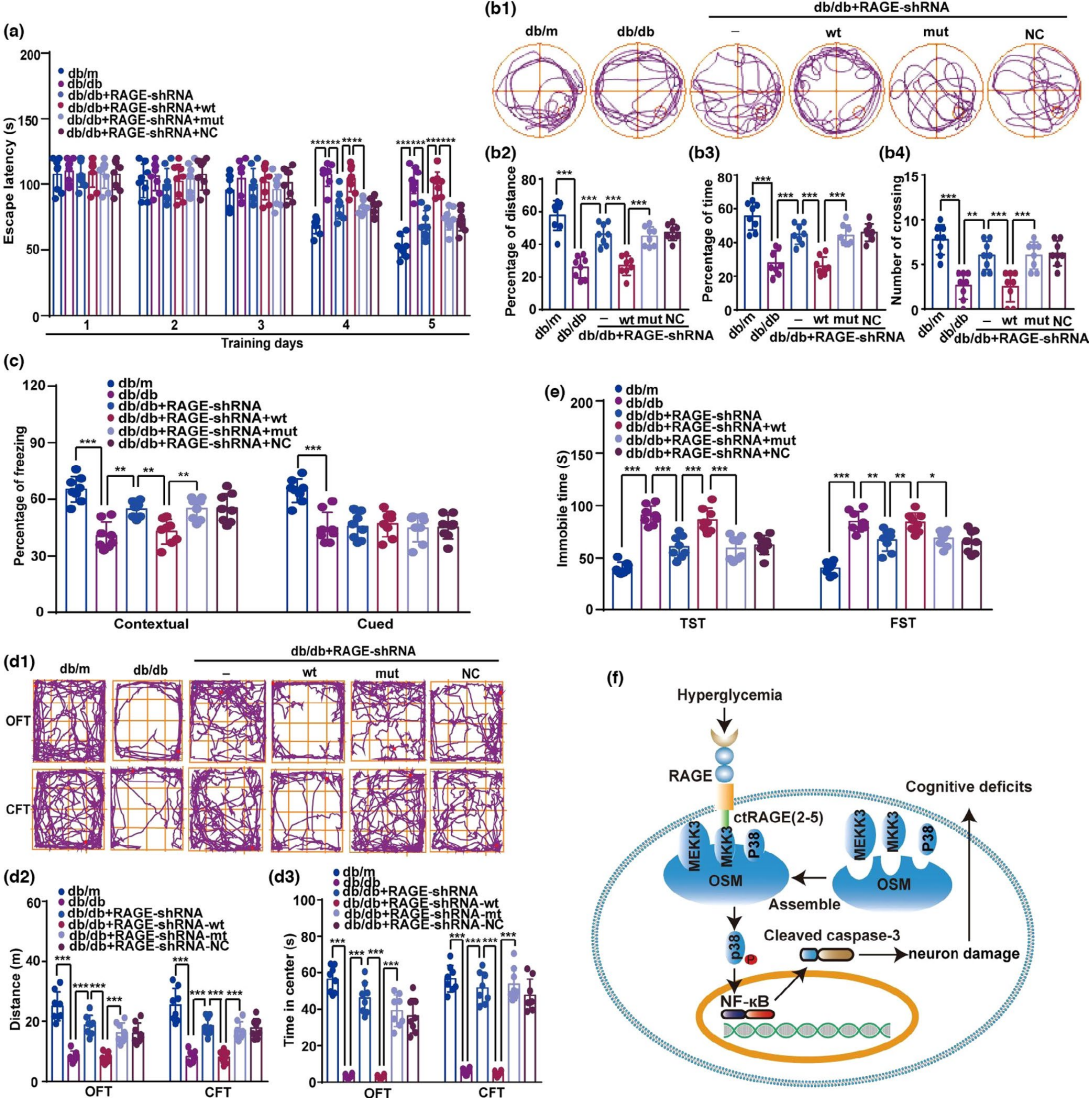
RAGE mutation improves diabetic encephalopathy in db/db mice. (a) Average escape latency for mice to reach the hidden platform on five consecutive training days was calculated. Data were analyzed using two‐way ANOVA and repeated measures followed by Sidak's test. For db/db compared with db/m, db/db + RAGE‐shRNA compared with db/db, db/db + RAGE‐shRNA + wt compared with db/db + RAGE‐shRNA, and db/db + RAGE‐shRNA + mut compared with db/db + RAGE‐shRNA + wt, *p* < 0.001. q (8, 120) = 8.325 (db/m versus db/db), 4.291 (db/db versus db/db + RAGE‐shRNA), 3.711 (db/db + RAGE‐shRNA versus db/db + RAGE‐shRNA + wt), and 3.969 (db/db + RAGE‐shRNA + wt versus db/db + RAGE‐shRNA + mut) on the 4th day, respectively. q (8, 40) = 11.750 (db/m versus db/db), 7.389 (db/db versus db/db + RAGE‐shRNA), 7.131 (db/db + RAGE‐shRNA versus db/db + RAGE‐shRNA + wtt) and 6.034 (db/db + RAGE‐shRNA + wt versus db/db + RAGE‐shRNA + mut), respectively, on the 5th day. ***p < 0.001. *n* = 8 in each group. (b1) Track maps of the probe trial on the 6th day without the platform. (b2–b4) The ratio of distance and time spent in target quadrant when the platform was taken away, and the number of the platform crossings during probe trial were evaluated on the sixth day. Data were analyzed by one‐way ANOVA followed by Tukey's test. *F* (5, 42) = 28.31 (B2), 25.44 (B3), and 14.90 (B4), respectively. ***p < 0.001, **p < 0.01 (db/db compared with db/db + RAGE‐shRNA for number of crossing). *n* = 8 in each group. (c) Freezing time ratio for the contextual and cued fear conditioning were assessed. One‐way ANOVA followed by Tukey's test was used. *F* (4, 35) = 21.29 in contextual fear conditioning, while *F* (4, 35) = 11.54 in cued fear conditioning. ***p < 0.001, **p < 0.01. In contextual fear conditioning, db/db + RAGE‐shRNA + wt compared with db/db + RAGE‐shRNA, *p* = 0.0039, db/db + RAGE‐shRNA + wt compared with db/db + RAGE‐shRNA + mut, *p* = 0.0031. *n* = 8 in each group. (d1) The track maps of mice in OFT and CFT. (d2 and d3) Total distance traveled and the time in the center were shown. Data statistics were performed using one‐way ANOVA followed by Tukey's test. *F* (5, 42) = 33.11 (OFT) and 31.65 (CFT) in distance. *F* (5, 42) = 83.52 (OFT) and 100.60 (CFT) in the number in center. ***p < 0.001. *n* = 8 in each group. (e) Immobility times in the TST and FST of mice were shown. Data were analyzed by one‐way ANOVA followed by Tukey's test. *F* (5, 42) = 30.08 (TST) and 25.05 (FST), respectively. ***p < 0.001. In FST, db/db compared with db/db + RAGE‐shRNA, *p* = 0.0049. db/db + RAGE‐shRNA compared with db/db + RAGE‐shRNA + wt, *p* = 0.0085. db/db + RAGE‐shRNA + wt compared with db/db + RAGE‐shRNA + mut, *p* = 0.0207. *n* = 8 in each group. (f) Graphical abstract of the mechanism underlying RAGE regulation of DE

Finally, we investigated the effect of specific mutation of RAGE on diabetic encephalopathy in db/db mice. There were no differences among the groups in the first 3 days of the acquisition phase in the MWM (Figure 8a), but the escape latency of db/db mice was longer than that for db/m mice on Days 4 and 5. On Days 4 and 5, db/db mice with mutant ctRAGE had shorter escape latencies than db/db mice with wild‐type ctRAGE (Figure 8a). Mice injected with mutated ctRAGE spent significantly more time and distance in the target quadrant and had a significantly greater number of platform crossings than mice with wild‐type ctRAGE (Figure 8b1–b4). As shown in Figure 8c, db/db mice displayed shorter freezing times than db/m mice in contextual and cued fear conditioning tests. In the contextual fear conditioning test, mice with mutant ctRAGE had significantly greater freezing time than mice with wild‐type ctRAGE.

Consistent with a previous study (Fourrier et al., 2019), in OFT and CFT, diabetic mice exhibited a decrease in the total distance traveled and time spent in the center compared with control mice (Figure 8d1). Decreases in total travel distance and time spent in the center were observed in the db/db + RAGE‐shRNA + wt group compared with the db/db + RAGE‐shRNA group, but locomotor activity was rescued in the db/db + RAGE‐shRNA + mut mice (Figure 8d1–d3). Similarly, mutated ctRAGE improved depression‐like behavior in db/db mice but wild‐type ctRAGE had no significant effect (Figure 8e). Collectively, these findings demonstrate that interruption of RAGE binding to MKK3 due to mutation in ctRAGE alleviates diabetic encephalopathy in db/db mice.

## 3 DISCUSSION

Our study identifies the precise molecular mechanism by which RAGE modulates the p38MAPK pathway in DE: Direct binding of ctRAGE AAs 2‐5 to MKK3 promotes assembly of the MEKK3‐MKK3‐p38 signaling module, which is critical for activation of the p38MAPK/NF‐κB pathway, ultimately exacerbating the development and degree of DE (Figure 8f). This is the first study to elucidate the mechanism underlying RAGE‐induced aggravation of DE. We also demonstrated that mutation of ctRAGE is sufficient to interrupt both RAGE binding to MKK3 and RAGE activation of the p38MAPK/NF‐κB signaling pathway, as well as to diminish diabetes‐induced neural damage and behavioral impairments.

Type 2 diabetic patients with mild cognitive impairment have increased serum levels of AGEs and their receptor, RAGE, indicating that the AGE‐RAGE system is a potential contributor to the development of cognitive decline in diabetes (Gorska‐Ciebiada et al., 2015). Activation of the p38MAPK/NF‐κB pathway by RAGE plays a pivotal role in the pathogenesis of nervous system complications in diabetes (Zhao et al., 2018). Over‐expression of RAGE has been shown to activate p38MAPK and NF‐κB signaling in the brains of diabetic mice, which may lead to neuronopathies and neuroinflammation, and ultimately cognitive dysfunction (Gorska‐Ciebiada et al., 2015; Zhao et al., 2018; Zhu et al., 2020). Hyperglycemia is linked to the formation of AGEs and the generation of reactive oxygen species (ROS), both of which are considered as major contributors to the development of cognitive deficits of diabetes (Momeni et al., 2021b). Interactions between AGEs and RAGE give rise to a cascade of events that triggers sustained activation of p38MAPK and NF‐κB signaling pathway, and further upregulation of RAGE and ROS production, ultimately leading to diabetes‐induced neurological complications (Momeni et al., 2021a; Tan et al., 2015). In contrast to wild‐type mice, neuronal synaptic plasticity and cognition were relatively well preserved in RAGE^−/−^ mice because of inhibition of the AGEs‐RAGE‐dependent signal transduction pathway (Momeni et al., 2021a; Zhang et al., 2014). Furthermore, the RAGE‐specific inhibitor FPS‐ZM1 reverses the effects of the AGEs‐RAGE system in high‐glucose conditions and dampens p38MAPK and NF‐κB signals (András et al., 2020; Tan et al., 2015; Xie et al., 2013), and ameliorates cognitive dysfunction in STZ‐induced hyperglycemic mice (Momeni et al., 2021b). Our present study identifies the detailed molecular mechanism by which RAGE activates p38MAPK/NF‐κB signaling, thus provides new information on the pathogenesis of DE. In the current study, inhibitory effect of FPS‐ZM1 on AGEs may be due to a reduction in the level of RAGE, which in turn leads to a reduction in intracellular fructose, glucose‐6‐phosphate, and glyceraldehyde‐3‐phosphate, which are the main components of AGEs, ultimately decreasing total AGEs levels. Studies to further investigate the mechanism by which FPS‐ZM1 inhibits AGEs are needed.

OSM acts as a scaffold for the assembly of MEKK3, MKK3, and p38 and is an obligate requirement for p38 phosphorylation in response to sorbitol in HEK‐293T cells (Uhlik et al., 2003). The link between MEKK3‐MKK3‐p38 and OSM is essential for the phosphorylation of p38MAPK, which directly affects cell apoptosis (Bai et al., 2016). Moreover, OSM is required for p38 activation in response to sorbitol: short interfering RNA knockdown of OSM or MEKK3 significantly decreased the signaling‐module‐dependent activation of p38 (Huth et al., 2016; Uhlik et al., 2003). Critically, we found that MKK3 binds RAGE directly, hinting that MKK3 is the target protein for RAGE. However, we did not exclude the possibility of a RAGE‐p38 interaction, which could affect RAGE upregulation of p38MAPK/NF‐κB signaling on a relatively small scale.

The membrane‐proximal domain of ctRAGE is also required for the direct interaction between RAGE and extracellular signal‐regulated kinase (Rai et al., 2012). What is the crucial motif that enables RAGE to bind directly to MKK3? It has been proposed that human ctRAGE contains an unusual α‐turn that mediates RAGE binding to mammalian diaphanous‐related formin (mDia1) through the ctRAGE residues Q364, R365, R366, and Q367, which correspond to R362, K363A, R364A, and Q365 in mouse ctRAGE (Rai et al., 2012). RAGE‐mDia1 interactions are required for RAGE‐dependent induction of a series of cellular processes (Ishihara et al., 2003). Although human ctRAGE and mouse ctRAGE are not 100% identical, they are similar to each other both in sequence and in structure (Rai et al., 2012). Our study of surface‐protein docking shows that MKK3, R175, and K329 contain both donors and acceptors of hydrogen bonds, which may be the reason that they are the central residues in the ctRAGE‐MKK3 interaction. The vacuum electrostatics of the surface‐containing K329 in MKK3 may be important for recognizing the RKRQ motif in ctRAGE, which maintains the unique binding conformation of ctRAGE‐MKK3. The AAs 23‐25 motif in ctRAGE, although not so important for the ctRAGE‐MKK3 interaction, is critical for the binding of chemical compounds with RAGE (Zhou et al., 2018). Our study suggests that mouse ctRAGE AAs 2‐5 is the key motif for RAGE binding to MKK3. These results are consistent with the report that without the last 18 residues of ctRAGE form the critical motif for ligand‐induced RAGE mediation of MAPK signals (Jules et al., 2013).

Protein–protein interactions are functionally relevant to the pathogenesis of many neurological disorders, such as stroke and Alzheimer's disease (AD; Jiang et al., 2020; Meng et al., 2018; Schulien et al., 2020; Zhong et al., 2018). Thus, there is growing interest in targeting protein–protein interactions for drug development (Dondelinger et al., 2019; Meng et al., 2018; Schulien et al., 2020; Zhong et al., 2018). Protein mutation that abrogates downstream signaling can suppress apoptosis and reduce inflammatory responses (Dondelinger et al., 2019; Meng et al., 2018; Schulien et al., 2020; Zhong et al., 2018). This is consistent with our finding that mutation of ctRAGE AAs 2‐5 is neuroprotective in db/db mice; the mutation prevents RAGE from interacting with MKK3, blocking activation of the p38MAPK/NF‐κB pathway.

Individuals with type 2 diabetes have a higher incidence of dementia, depression, and anxiety, which together are considered characteristic manifestations of DE (Raffield et al., 2016; van Sloten et al., 2020). Prolonged exposure to hyperglycemia leads to a series of structural and functional changes in some brain regions (Chen et al., 2018). Animal studies suggests that glucose levels are up to three times greater in the hippocampus of diabetic rats compared with control ones (Toth et al., 2007). Thus, the hippocampus, which is a key region for learning, memory, and emotion regulation (Chen et al., 2019; Lopes et al., 2020; Ripoli et al., 2020), appears to be particularly susceptible to hyperglycemia (Gilbert et al., 2019).

The present work demonstrates that mutation of ctRAGE ameliorates neuronal loss, as well as rescues morphological changes and LTP in hippocampal CA1 subarea. Furthermore, ctRAGE mutation decreases PPR in db/db mice, indicating that changes in hippocampal synaptic plasticity involve both pre‐ and postsynaptic changes, as previously shown in diabetic animal models (Momeni et al., 2021a; Trudeau et al., 2004). Both NMDAR and AMPAR function, which are essential for induction and maintenance of LTP, are regulated by RAGE and the related p38MAPK signaling pathway (Huang et al., 2004; Momeni et al., 2021a; Stein et al., 2021). Here, we confirm that mutation of ctRAGE may change the subunits and/or trafficking of AMPARs, but has no effect on ionotropic NMDARs. This difference in receptor responses to ctRAGE mutation produced an increase the AMPAR/NMDAR ratio, an important feature of synaptic LTP (Ma et al., 2018). However, whether non‐ionotropic NMDA receptor signaling is modulated by ctRAGE mutation requires further investigation. We also showed that morphological and functional changes in hippocampal neurons and cognitive impairments could be alleviated by ctRAGE mutation. It is likely that ctRAGE AAs 2‐5 is the crucial RAGE motif, and plays a pivotal role in RAGE‐accelerated DE.

It is well documented that over‐expression of RAGE participates in the pathogenesis of Parkinson's disease (PD) by activating the p38MAPK/NF‐κB signaling cascade (Wang et al., 2020). The activation of p38MAPK intracellular inflammatory pathways in neurons is known to be involved in the LTP impairments and cognitive deficits caused by AD (Liu et al., 2012; Origlia et al., 2008; Takuma et al., 2009), as well as in stroke‐induced neuronal cell death (Lok et al., 2015). Blocking RAGE from activating p38MAPK may thus alleviate neuronal dysfunction in PD, AD, and stroke. However, how RAGE mediates p38MAPK signaling pathway remains to be fully elucidated. The data in the present study indicate that ctRAGE AAs 2‐5 is the crucial motif for RAGE modulation of p38MAPK pathways; mutation of ctRAGE blocks p38MAPK/NF‐κB activation in hippocampus. Thus, these data provide important evidence that ctRAGE AAs 2‐5 may serve a potential therapeutic target for neurodegenerative diseases such as PD, AD, and stroke.

In summary, our study demonstrates that binding of ctRAGE AAs 2‐5 to MKK3 is an essential event for MEKK3‐MKK3‐p38 assembly and activation of the p38MAPK/NF‐κB pathway under high‐glucose conditions. Our findings cement the notion that targeting ctRAGE AAs 2‐5 may facilitate neuroprotection and yield effective and innovative drugs for therapy against DE.

## 4 METHODS AND MATERIALS

### 4.1 Antibodies and reagents

All antibodies, chemicals, recombinant proteins, critical commercial assays, cell lines, experimental models, oligonucleotides, and recombinant DNA are listed in Table S1.

### 4.2 Cell‐lines experimental design

HEK‐293T and HT22 cell lines were cultured and maintained in Dulbecco's modified Eagle's medium supplemented with 10% fetal bovine serum (FBS) at 37°C with 20% O_2_ and 5% CO_2_, and 0.05% trypsin/EDTA was used for cell passaging. As in our previous study (Ying et al., 2021), 25 and 80 mM glucose were used for the normal‐ and high‐glucose conditions in HEK293T cells experiments. After 12 h in serum‐free culture for synchronization, HEK‐293T cells were randomly divided into five groups: a normal group (NG; 25 mM glucose), a high‐glucose group (HG: 80 mM glucose), a high‐glucose plus RAGE inhibitor group (FPS; 2.5 μM FPS‐ZM1 dissolved in DMSO), a high‐glucose plus solvent control group (DMSO), and an osmotic pressure control (mannitol) group (MG; 25 mM glucose plus 55 mM mannitol). Cells were collected 24 h after high‐sugar stimulation. Some HEK‐293T cells were also transfected with MKK3‐shRNA or nonsense control plasmids. After 24 h, these cells were synchronized, subjected to high glucose, and collected.

HT22 cells require 25 mM basal glucose for optimal growth and survival, and 50 mM or even higher glucose concentrations are usually used for high‐glucose conditions (Liu et al., 2016, 2017; Zhang et al., 2020; Zhang et al., 2020). HT22 cells were transfected with LV‐RAGE‐shRNA to knock down RAGE expression. Five to seven days after transfection, stable RAGE‐knockdown cells (RAGE‐shRNA) were divided into four groups: a high‐glucose group (HG + RAGE‐shRNA; 50 mM glucose), a group transfected with LV‐Flag‐RAGE‐wt (to over‐express wild‐type ctRAGE) and then treated with high glucose (HG + RAGE‐shRNA + wt), a group transfected with LV‐Flag‐RAGE‐mut (to over‐express ctRAGE with the R2A‐K3A‐R4A‐Q5A mutation) and then treated with high glucose (HG + RAGE‐shRNA + mut), and a group transfected with a nonsense control (LV‐NC) and then treated with high glucose (HG + RAGE‐shRNA + NC). Before stimulation, cells were transferred to serum‐free DMEM for synchronization. Cells were harvested 24 h after high‐sugar stimulation.

### 4.3 Mice: experimental design

C57BL/6 mice (6 weeks old, 18–20 g) were fed with a high fat diet (HFD) for 8 weeks; then, a diabetic model was induced by injecting 40 mg/kg STZ (dissolved in 0.1 M citrate phosphate buffer, intraperitoneal injection (i.p.)) daily for three consecutive days. After 3 days, mice with random blood glucose levels higher than 16.7 mM/L were confirmed as diabetes (Zhang et al., 2020; Zhang et al., 2020; Zhao et al., 2021). Diabetic mice were randomly assigned to a diabetes group (DM), a DM plus RAGE inhibitor group (FPS; FPS‐ZM1 1.0 mg/kg dissolved in corn oil, 3.33 ml/kg/day, i.p.; Deane et al., 2012; Fan et al., 2020), or a DM plus corn oil group (Oil; 3.33 ml/kg/day, i.p.). C57BL/6 mice of the same age served as a control group (Con). FPS‐ZM1 was administered once daily for 8 weeks.

Male db/db mice (6–8 weeks old) were assigned into a db/db group (db/db), a db/db plus FPS‐ZM1 group (FPS), or a db/db plus corn oil group (Oil); age‐matched db/m mice were used as a control. The experimental design was the same as for the STZ‐induced diabetic mice. In a second experiment, 6‐ to 8‐week‐old db/db male mice received bilateral micro‐injection of LV‐RAGE‐shRNA in the hippocampus to knock down RAGE (db/db + RAGE‐shRNA). Two weeks later, RAGE‐knockdown db/db mice were injected intracranially with either LV‐RAGE‐wt to over‐express wild‐type ctRAGE (db/db + RAGE‐shRNA + wt), LV‐RAGE‐mut to over‐express ctRAGE with the R2A‐K3A‐R4A‐Q5A mutation (db/db + RAGE‐shRNA + mut), or with a nonsense control (db/db + RAGE‐shRNA + NC).

All mice were maintained under specific pathogen‐free conditions and housed in clear plastic cages on a 12 h/12 h light/dark cycle at 23 ± 1°C with free access to water and food. Animal experimental procedures were conducted in accordance with the guidelines described in the revised 2011 Chinese Regulations for the Administration of Affairs Concerning Experimental Animals and approved by the Institutional Animal Care and Use Committee of Xuzhou Medical University.

### 4.4 Cell transfection

Experiments were performed after 4‐5 passages. HEK‐293T cells were transfected with pCDNA3.1‐His‐MEKK3, pCDNA3.1‐His‐MKK3, pCDNA3.1‐His‐p38, and pCDNA3.1‐His‐OSM when cell confluence reached 80%, using EL according to the protocol from the manufacturer. After incubation with the plasmids, cells were treated with normal glucose for 48 h. HT22 cells were first transfected with LV‐RAGE‐shRNA. Stable knockdown cells were transfected with LV‐Flag‐RAGE‐wt/mut (to over‐express either wild‐type ctRAGE or ctRAGE with the R2A‐K3A‐R4A‐Q5A mutation). The procedure was performed according to the manufacturer's instructions, and the medium was replaced with fresh complete medium 24 h after transfection. Then, the culture medium containing puromycin (6 μg/ml) or neomycin (400 μg/ml) was replaced every day until all uninfected cells had died. After 5–7 days, the stable cells were obtained. From then on, transfected cells were cultured in complete medium.

### 4.5 Flow cytometry analysis

Apoptosis rate was determined by the Annexin V‐PE/7‐amino‐actinomycin D (7‐AAD) Apoptosis Detection Kit. Briefly, cells were collected with trypsin without EDTA, and 1 × 10^6^/100 μl of cells were placed in a flow tube and washed and suspended in PBS three times. Then, 50 μl of bindbuffer and 5 μl of Annexin V were added into each flow tube. After 15 min, 1 μl of 7‐AAD was applied, and the tubes were placed in a dark room for 15 min. The apoptotic rate was detected by flow cytometry, and data were processed in FlowJo software.

### 4.6 Enzyme‐linked immunosorbent assay

AGEs and soluble sRAGE in conditioned media and hippocampal tissue homogenates were measured using Enzyme‐linked immunosorbent assay Kit according to the manufacturer's instructions. Cell supernatants were obtained by centrifugation (1000 *g*, 10 min). Hippocampal samples were diluted with PBS followed by homogenization and then centrifugated at 1000 g for 10 min. 96‐well plates were coated with test liquid A for 1 h, then were washed three time with PBS and incubated with test liquid B for 30 min. After washing five times, samples were incubated in the dark in 50 μl substrate for 20 m, and the reaction was blocked with 50 μl stop buffer. Absorbance was read at 450 nm on a Spectra Max Plus plate reader. Experiments were carried out three times on each sample.

### 4.7 Oral glucoset olerance test and water consumption

An oral glucose tolerance test (OGTT) was performed in STZ‐induced diabetic mice (24 weeks old) and db/db mice (17–19 weeks old). After overnight fasting, mice were orally administered glucose (2 g/kg body weight), and tail–vein blood samples were collected at 0, 0.5, 1, and 2 h after glucose treatment. Next day, the water intake of mice was monitored for 24 h.

### 4.8 Intra‐hippocampal injections

Male 6‐ to 8 week‐old db/db mice were microinjected with LV‐RAGE‐shRNA. First, mice were anesthetized with 1.5% pentobarbital (0.6 ml/g) and then fixed in a stereotaxic frame. Mice were injected with 1 μl LV‐RAGE‐shRNA or LV‐NC into the hippocampal CA1 subregion bilaterally at a location of AP −2.0 mm, ML ±1.7 mm, and DV −2.2 mm. The injection velocity was 0.25 μl/min, and the micro‐injector was kept in place for 5 min after the injection. Two weeks later, RAGE knockdown was confirmed and mice were then injected with LV‐Flag‐RAGE‐wt or mut, following the same procedures.

### 4.9 Protein collection and extraction

For cellular protein, cells were washed with cold PBS and harvested by centrifugation at 120 *g* for 10 min. For animal protein, mice were anesthetized; then, the hippocampi were dissected on ice, and immediately frozen in liquid nitrogen and stored at −80°C. Nuclear and cytoplasmic proteins were extracted with cytoplasmic and nuclear protein extraction Kits. Briefly, samples were resuspended in three pellet volumes of cold homogenization buffer A (20 mM HEPES, pH 7.0, 0.15 mM EDTA, 0.015 mM EGTA, 10 mM KCl, 1% NonidetTM P40, 1 mM phenylmethylsulphonyl fluoride, 20 mM NaF, 1 mM sodium pyrophosphate, 1 mM sodium orthovanadate, and 1 mg/ml leupeptin) containing phenylmethanesulfonyl fluoride (PMSF) and incubated on ice for 30 min. The homogenate was centrifuged at 500 *g* for 5 min, and the supernatants were taken as the cytosolic fraction. The nuclear pellet was resuspended in five volumes of cold homogenization buffer B (10 mM HEPES with pH 8.0, 0.1 mM EDTA, 0.1 mM NaCl, 25% glycerol, 1 mM phenylmethylsulphonyl fluoride, 20 mM NaF, 1 mM sodium pyrophosphate, 1 mM sodium orthovanadate, and 1 mg/ml leupeptin). After centrifugation, supernatants were collected as the nuclear fraction. Proteins were quantified with a BCA Protein Assay Kit. Protein concentrations were calculated according to the Lowry method.

### 4.10 Immunoprecipitation

Protein samples were diluted with 400 μl immunoprecipitation buffer (50 mM/L HEPES with pH 7.1, 10% glycerol, 150 mM/L NaCl, 1 mM/L ZnCl_2_, 1.5 mM/L MgCl_2_, 1% Triton X‐100, 0.5% NP‐40, and the phosphatase and protease inhibitors listed for homogenization buffer A). The diluted protein samples were incubated with the indicated primary antibodies or negative control IgG overnight at 4°C. The next day, protein A/G‐Agarose was added to the mixture for 2 h at 4°C. The complex was washed 5 times via centrifugation at 1000 *g* for 2 min at 4°C with immunoprecipitation buffer. The supernatants were subjected to immunoblotting.

### 4.11 Immunoblotting

Equivalent amount of proteins mixed with 4× loading buffer were separated by SDS‐PAGE and then transferred electrophoretically to an NC membrane (Amersham Biosciences). After blocking with 3% bovine serum albumin, the membranes were incubated first with the appropriate primary antibodies for 16 h at 4°C. The next day, the membranes were washed twice in Tris‐buffered saline Tween 20 for 5 min and then with the appropriate secondary antibodies for 2 h. Immunoreactive bands were scanned with an Odyssey Laser imaging scanner (Olympus FV10i) and analyzed in Image J.

### 4.12 Immunofluorescence

For the co‐localization of RAGE and MKK3, fixed cells were permeabilized with PBS containing 0.3% Triton X‐100 for 30 min and then blocked with 1% goat serum for 1 h. Cells were incubated with the appropriate primary antibodies (RAGE and MKK3), overnight at 4°C. After rinsing three times with PBS, cells were incubated with AlexaFluor 488‐conjugated rabbit or AlexaFluor 594‐conjugated mouse secondary antibodies (1:1000) for 2 h and then stained with DAPI for 7 min. Cells were imaged with a confocal microscope (Zeiss), and the overlap value (the ratio of fluorescence co‐localization) was calculated.

For NeuN immunohistochemistry, mice were perfused with 0.9% saline, followed by 4% paraformaldehyde. The brains were removed, postfixed overnight, and dehydrated in 30% sucrose in 0.1 M PBS. Brain tissue was cut longitudinally into 20‐μm sections with a cryostat and mounted on slides. All hippocampal sections were permeabilized with 0.3% Triton X‐100 in PBS for 30 min and then blocked with a non‐specific IgG followed by incubation with the appropriate primary (NeuN) antibodies for 16 h at 4°C. The next day, the sections were incubated with AlexaFluor 488‐conjugated mouse secondary antibodies (1:1000) in a dark environment for 2 h. Finally, sections were incubated with DAPI for 7 min and were observed with a confocal microscope (Zeiss). DAPI^+^ and NeuN^+^ neurons were considered as surviving, and surviving pyramidal neurons in the hippocampal CA1 subregion per 1 mm length were counted as the neuronal number.

### 4.13 Docking methods

MKK3 model building was accomplished by homology model‐building online with Swiss‐Model (Waterhouse et al., 2018), using a previously reported MKK6 model (PDB: 3VN9) with an MKK3 sequence (Mus musculus), since MKK3 closely resembles MKK6 in amino acid sequence. The C‐terminal of RAGE (Mus musculus, residues 362–402) was built in a random conformation by Coot (Emsley et al., 2010). Docking experiments were performed with Autodock Vina (version 1.1.2; Trott & Olson, 2010) Amino acids in the C‐terminal of RAGE were set as flexible residues, and all bonds were set as rotatable except the peptide bonds. A genetic algorithm was used to minimize system energy and prepare docking conformations using the default settings. A total of 50 possible substrate conformations were exported by docking. The conformations that were consistent with the pull‐down experiment were extracted. The conformations with maximum docking score were used for subsequent analysis. The model figures representing protein interaction were drawn in PyMOL (version 0.99).

### 4.14 Golgi staining and structural analysis

Golgi staining with an FD rapid golgi stain Kit was performed to assess dendritic intersections and dendritic spines of pyramidal neurons in the CA1 subfield. Unfixed brains were removed from the skull quickly and immersed in specific impregnation solution containing equal parts of solution A and B at room temperature under dark conditions for 14 days, then incubated in solution C at 4°C for 72 h. Brain tissue was serially sectioned into 100 µm coronal sections with a vibratome and attached with solution C to a 0.5% gelatin‐coated glass slide. After sections were rinsed in distilled water, a mixed 1:1:2 solution consisting of solution C, solution D, and distilled water was employed to stain the sections for 5 min. Sections were then dehydrated in 70% alcohol for 10 min, 90% alcohol for 10 min, 100% alcohol for 20 min, and xylene for 20 min. Finally, the sections were embedded with neutral balsam and cover glasses and observed with a Leica microscope.

Dendritic crossings were analyzed with Sholl analysis. The count of dendritic intersections of pyramidal neurons in the CA1 subfield included apical and basal dendrites. Neurons that met the following characteristics were analyzed: (1) Neurons were relatively isolated neighboring cells to allow unambiguous identification of the dendritic tree; (2) neurons had good staining and impregnation without breaks; (3) neurons had mainly intact and fully impregnated apical and basal arborizations without truncated branches. The number of dendritic intersections was counted in successive radial segments 10–120 μm from the neuronal soma. Second‐order basilar dendritic branches with clear spine resolution were selected arbitrarily in basal or apical dendrites, and the number of dendritic spines per 10 μm was counted in Image J.

### 4.15 GST pull‐down assay

The vector pGEX‐4T‐1 was used to express RAGE and two different ctRAGE (R2A‐K3A‐R4A‐Q5A and R23A‐E25A) mutations. GST and GST‐tagged wild‐type/mutant RAGE were expressed in BL21 cells and then purified with a GST protein interaction pull‐down Kit. HEK‐293T cells over‐expressing His‐tagged MEKK3, MKK3, p38, and OSM proteins in vector pcDNA3.1 were lysed in homogenization buffer. The GST pull‐down experiment was performed according to the manufacturer's instructions. Briefly, 50 µl of garose resin was pre‐equilibrated in TBS containing the pull‐down lysis buffer. GST‐tagged protein (200 µl) was incubated with the resin at 4°C for 2 h on a rotating platform. After washing five times in TBS containing the pull‐down lysis buffer, the MEKK3, MKK3, p38, and OSM reaction products were co‐incubated with GST‐tagged protein for 2 h. Finally, the bound proteins were eluted and subjected to immunoblotting.

### 4.16 Behavioral experiments

Behavioral testing began when mice were aged 23 weeks (STZ‐induced diabetic mice) or 16–18 weeks (db/db mice). The sequence of tests was as follows: MWM, fear conditioning, OFT, CFT, TST, and FST. Behavioral tests were analyzed with the ANY maze video tracking system (Stoelting) with a CCD (Charge Coupled Device). There was an interval of 24 h between each experiment.

#### 4.16.1 Morris water maze

The test was performed over the course of 6 consecutive days: 5 days of training and 1 day with a probe test. The MWM test was conducted in a dark room. A circular pool 120 cm in diameter was filled with opacified (non‐toxic white paint) water. The depth of water was 30 cm, and the water temperature was 22–26°C. The pool was divided into four symmetrical quadrants with an invisible escape platform hidden 1 cm beneath the surface of the water in a fixed quadrant. During the acquisition phase, each mouse was subjected to four swimming trials on each of 5 training days. On the training days, the mouse was placed into the water from four quadrants orderly with facing the outer edge of the pool. The mouse was required to explore the hidden platform for 120 s. If the mouse found the hidden platform before the end of the 120 s, it was allowed to remain on the platform for 10 s. If the mouse failed to find the platform in 120 s, it was guided manually to the platform and allowed to remain there for 10 s, and the escape latency was regarded as 120 s. On the sixth day, the platform was removed and mice swam freely for 120 s. For this single probe trail, the number of times the mouse crossed the prior position of the platform, the ratio of time spent in the target quadrant, and the ratio of swimming distance spent in the target quadrant were recorded and analyzed.

#### 4.16.2 Fear conditioning

The training chamber used for fear conditioning was made from plexiglas with a stainless‐steel grid floor for shock delivery. First, a mouse was placed in the training chamber and allowed to explore the enclosure for 3 min. Next, three 30‐s tone stimuli (2000 Hz, 70 db) were delivered, accompanied by a shock (0.7 mA) during the last 2 s of the tone. The three tone–shock pairings were separated by 1 min. The mouse was then returned to the home cage, and the training chamber was cleaned with 95% ethyl alcohol. After 24 h, the mouse was returned to the training chamber for 8 min, without delivery of either tone or foot shock stimuli, and the ratio of freezing time to the total recorded time was recorded via a camera as a measure of contextual fear conditioning. Two hours later, the mouse was placed in a novel chamber without a stainless‐steel floor grid. After a 3‐min exploratory period in this new chamber, the 70‐db tone was presented for 3 min and the ratio of freezing time to total recorded time was used as a measure of fear conditioning to the cue.

#### 4.16.3 Open field test and closed field test

In the OFT, a mouse was placed into the center of an open field (W50 × D50 × H30 cm), in which the floor was demarcated into 16 squares. After a 3‐min adaptation period, the mouse explored freely. The CFT was performed in a closed field apparatus of the same size as the open field chamber. Mice were placed in the center field. After a 3‐min adaptation period, mice were allowed to explore freely. The total distance traveled and the time spent in the center (defined as the central four squares) were recorded over a 5‐min period for both the OFT and the CFT.

#### 4.16.4 Tail suspension test and forced swim test

In the TST, animals were suspended 40 cm above the ground by the tail. After a 2‐min adaptation period, the total time spent immobile during a 4‐min period was recorded. In the FST, mice were placed in a clear glass cylinder 45 cm in height and 19 cm in diameter, filled with water to a depth of 23 cm, and maintained at a temperature of 22–25°C. After a 2‐min adaptation period, the total time spent immobile during a 4‐min period was recorded.

### 4.17 Electrophysiological recordings

The procedure was conducted as described previously (Hao et al., 2015; Huang et al., 2021; Ma et al., 2018). After decapitation, brains were rapidly removed and placed in ice‐cold artificial cerebrospinal fluid (ACSF; in mM: 126 NaCl, 2.5 KCl, 1 MgCl_2_, 1 CaCl_2_, 1.25 KH_2_PO_4_, 26 NaHCO_3_, and 20 glucose, 290–300 mOsm, pH 7.4) oxygenated with 95% O_2_ and 5% CO_2_. Transverse hippocampal slices of 320 μm thickness were cut with a Leica VT1000s vibratome (Leica Biosystems), then incubated with ACSF saturated with 95% O_2_ and 5% CO_2_ at 28°C for 1 h prior to the start of electrophysiological recordings.

fEPSPs were recorded with glass patch pipettes filled with ACSF positioned in the stratum radiatum of the CA1 subarea. A concentric electrode CBARB75 (FHC Inc.) was used to elicit synaptic responses by stimulating of the Schaffer collateral fibers. Single baseline stimulation was delivered with an intensity that elicited ~50% of the maximum amplitude at 0.05 Hz. LTP was induced by two consecutive 1‐s trains of 100‐Hz stimuli, with a 20‐s interval between trains. LTP was quantified by comparing the mean fEPSP slope during the baseline period with the mean fEPSP slope during the last 10 min of the recording period, and calculating the percentage change from baseline. The PPR of the fEPSP was defined as the percentage change in the amplitude of the second evoked response relative to the first with a 25‐ms interval between pulses.

For whole‐cell patch‐clamp recordings, glass patch pipettes were filled with a solution containing 140 mM K‐methylsulfate, 4 mM NaCl, 10 mM HEPES, 0.2 mM EGTA, 4 mM MgATP, 0.3 mM Na_3_GTP, and 10 mM phosphocreatine (pH was adjusted to 7.4 with KOH). To isolate glutamatergic currents, GABA_A_ receptors were blocked with bicuculline (10 μM). For AMPAR‐EPSC recordings, the holding potential was –65 mV. The input–output curves for AMPAR EPSCs were recorded by stimulating the Schaffer collateral‐CA1 pathway with gradually increasing intensities (0.1, 0.15, 0.2, 0.25, and 0.3 mA). For NMDAR‐mediated EPSC recordings, the holding potential was +40 mV. The peak currents were estimated 30 ms after the peak AMPAR EPSCs, when the contribution of the AMPAR component was minimal. The AMPAR/NMDAR ratio was calculated by dividing the mean NMDAR EPSC amplitude by the mean AMPAR EPSC amplitude. The intensity used for the NMDAR‐EPSC recordings and the calculation of AMPAR/NMDAR ratio was 0.2 mA.

Data were accepted when series resistance fluctuated within 15% of the initial values (15–25 MΩ).

All signals were amplified by an Axon‐700B amplifier (Molecular Devices), filtered at 2 kHz, and sampled at 10 kHz with a Digidata 1440 device (Molecular Devices). Traces were acquired with Clampex 10.2 and analyzed in Clampfit 10.2 software (Molecular Devices).

### 4.18 Statistical analysis

All statistical analyses were conducted in GraphPad Prism 7.0 software. Protein intensity, apoptosis rate, AGEs levels, immunofluorescence overlap value, dendritic spine number, electrophysiology data, weight change, blood glucose, and water intake data were analyzed with one‐way ANOVA followed by Tukey's test. The distance and time spent in the target quadrant, the number of the platform crossings, the total distance traveled, the time in the center, the total immobility time, and the freezing ratio were also analyzed with one‐way ANOVA followed by Tukey's test. Data from the MWM training sessions, weight and blood glucose changes in STZ‐induced diabetes, the number of dendritic intersections, the OGT, and AMPAR EPSCs were analyzed with two‐way ANOVA followed by Tukey's multiple comparisons test. Data are presented as the mean ±standard deviations (SD). Values of *p* < 0.05 were considered to be statistically significant.

## CONFLICT OF INTEREST

All authors declare no competing interests.

## AUTHORS’ CONTRIBUTIONS

Xiao‐Yan Zhou, An‐An Li, and Yuan‐Jian Song supervised this study. Xiao‐Yan Zhou, Chang‐Jiang Ying, Bin Hu, and Yuan‐Jian Song designed and analyzed all of the experiments. Xiao‐Yan Zhou and Tian Gan performed the experiments in Figures 1 and 2, Figures S1, and S2. Chang‐Jiang Ying, Tian Gan, Yusheng Zhang, Yan‐Dong Zhu, Bing Hu, and Nan Wang carried out the experiments in Figures 3 and 4. Xiao‐Yan Zhou, Chang‐jiang Ying, Yu‐Sheng Zhang, and Bing Hu conducted the experiments in Figures 5, 6, 8, Figures S3, S4, and S5. Bing Hu conducted the experiments in Figure 7. Xiao‐Yan Zhou and Bing Hu interpreted the data and wrote the manuscript together with An‐An Li.

### OPEN RESEARCH BADGES

This article has earned an Open Data, for making publicly available the digitally‐shareable data necessary to reproduce the reported results. The data is available at https://osf.io/9evyn/quickfiles.

## Supporting information

Supinfo S1

## Data Availability

All data generated and/or analyzed during this study are included in this article, and the data that support the results of this study are available from the corresponding author upon reasonable request.
