# Supplementary material for: Receptor for advanced glycation end products aggravates cognitive deficits in type 2 diabetes through binding of C‐terminal AAs 2‐5 to mitogen‐activated protein kinase kinase 3 (MKK3) and facilitation of MEKK3‐MKK3‐p38 module assembly
Source: Aging Cell. 2022 Jan 26;21(2):e13543. doi: 10.1111/acel.13543 (PMC8844116; doi:10.1111/acel.13543)
Supplement: Supplementary file 1 — Supinfo S1 [file ACEL-21-e13543-s001.pdf]

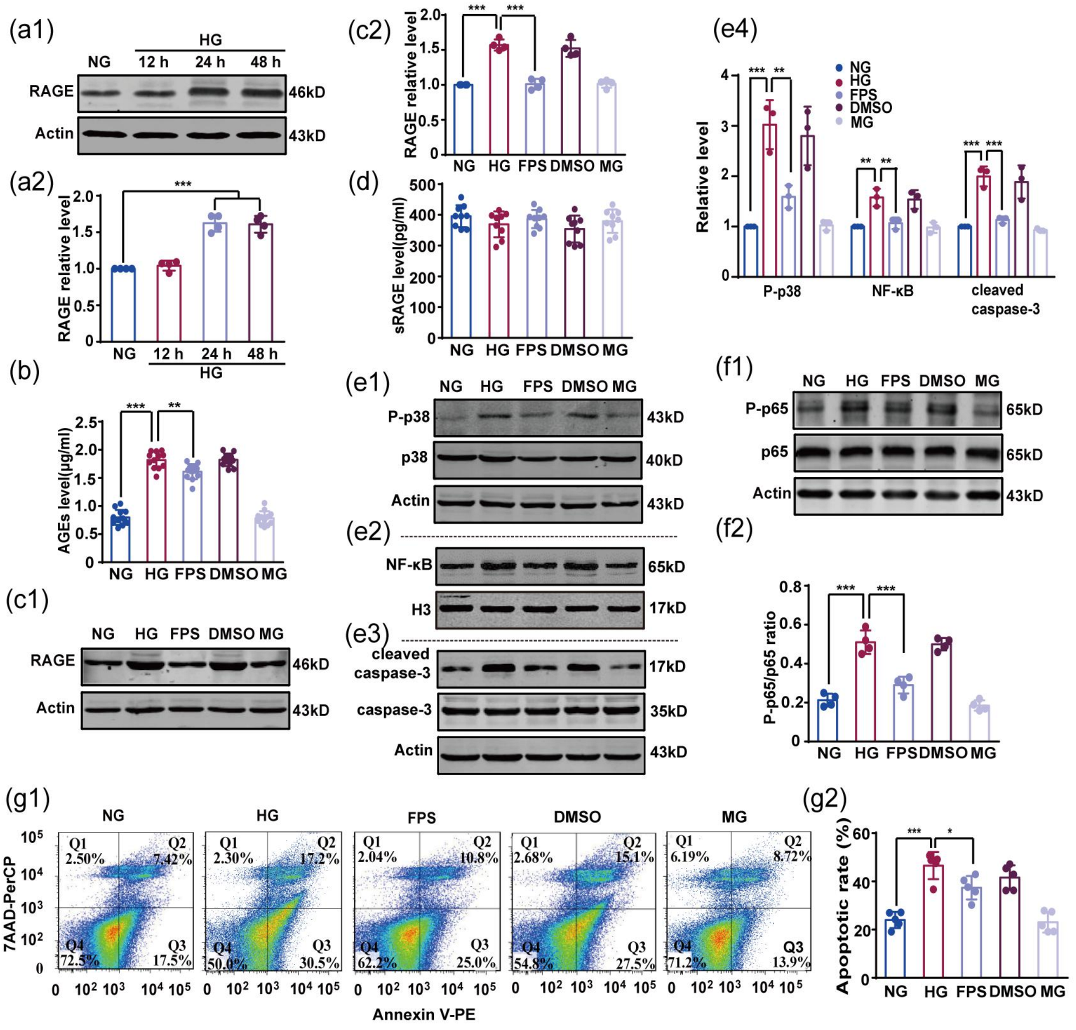

Supplementary Figure 1 Inhibition of the interaction between RAGE and the MEKK3-MKK3-P38 signaling module decreases activation of p38MAPK/NF-κB signaling pathway. (a1) RAGE levels with difference duration of high glucose condition were shown. **(a2)** Relative intensity was evaluated and presented as fold change to Con group. Data were analyzed by One-way ANOVA followed by Tukey's test. F (3, 12) = 60.29. $^{***}P < 0.001$. n = 4 in each group. **(b)** The AGEs level under high-glucose conditions were detected by ELISA. Data were analyzed by one-way ANOVA followed by Tukey's test. F (4, 55) = 197.80. NG compared with HG, $P <$ 0.001. HG compared with FPS, $P = 0.0029$. n = 12 in each group. **(c1)** The expression of RAGE in HEK-293T cells induced by high glucose was examined by immunoblotting with the anti-RAGE antibody. **(c2)** The relative intensity of RAGE was assessed. Data were analyzed by one-way ANOVA followed by Tukey's test. F (4,

15) = 57.35. $^{***}P < 0.001$. n = 4 in each group. **(d)** sRAGE level under high-glucose conditions was detected by ELISA. Data were analyzed by one-way ANOVA followed by Tukey's test. F (4, 40) = 1.60. n = 9 in each group. **(e1-e3)** The expression of P-p38 and cleaved caspase-3, and the level of NF-κB were detected by immunoblotting with the anti-P-p38, cleaved caspase-3 and NF-κB antibodies respectively. **(e4)** Analysis for optical density value of P-p38, NF-κB and cleaved caspase-3 were presented. Data were shown as fold change to NG group and analyzed by one-way ANOVA followed by Tukey's test, F (4, 10) = 22.08 (P-p38), 15.24 (NF-κB) and 25.75 (cleaved caspase-3) respectively. $^{***}P < 0.001$, $^{**}P < 0.01$. When HG compared with FPS in P-p38, $P = 0.0043$. In the level of NF-κB, NG compared with HG, $P = 0.0026$, HG compared with FPS, $P = 0.0062$. n = 3 in each group. **(f1 and f2)** Cytoplasmic phosphorylated p65 (P-p65) was detected and the ratio of P-p65/p65 was shown. Data were analyzed by one-way ANOVA followed by Tukey's test. F (4, 15) = 58.43. $^{***}P < 0.001$. n = 4 in each group. **(g1)** Typical cellular flow cytometry images of cellular flow cytometry displayed cellular apoptosis. Dead cells in Q1, late apoptotic cells in Q2, viable apoptotic cells in Q3, and normal cells in Q4. **(g2)** Apoptotic rate (number of cells in Q2 and Q3 / total number of cells) were shown. One-way ANOVA followed by Tukey's test was used. F = (4. 20) 25.02. $^{***}P < 0.001$, $^{*}P < 0.05$, HG compared with FPS, $P = 0.0428$. n = 5 in each group.

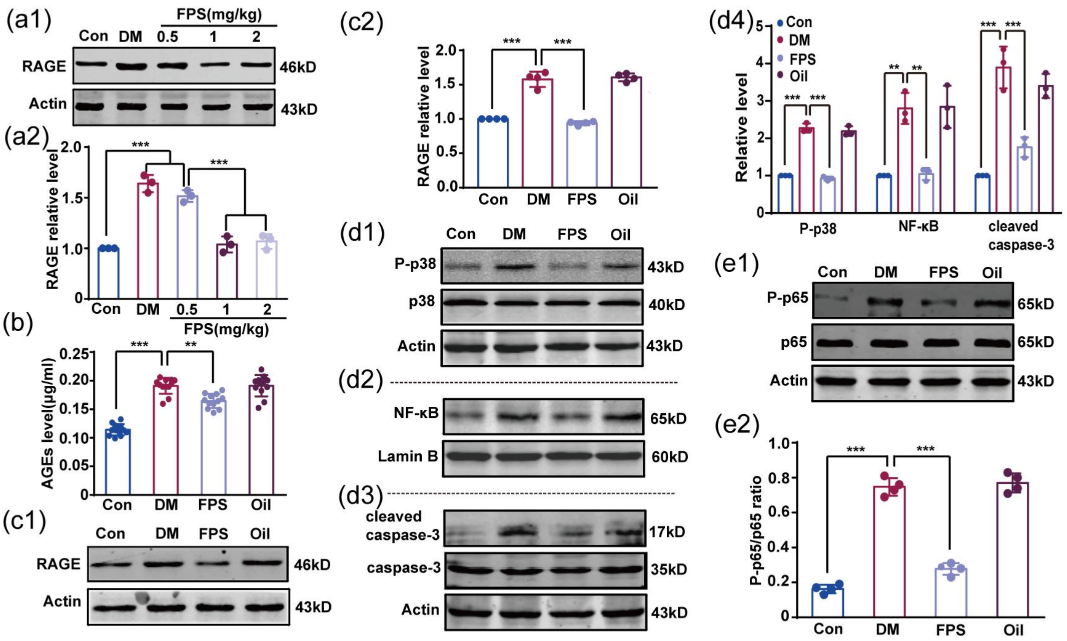

Supplementary Figure 2 RAGE co-precipitates with the MEKK3-MKK3-p38 signaling module and facilitates p38MAPK/NF-κB signal activation in hippocampus of diabetic mice. **(a1)** Effects of difference doses of FPS-ZM1 on RAGE levels were displayed. **(a2)** Optical intensity was assessed using optical density and presented as fold change to Con group. One-way ANOVA followed by Tukey's test. F (4, 10) = 61.38 $^{***}P < 0.001$. n = 3 in each group. **(b)** The AGEs level in hippocampus was tested by ELISA. Data were analyzed with One-way ANOVA followed by Tukey's test. F (3, 44) = 79.98. Con compared with DM, $^{***}P < 0.001$. DM compared with FPS, $P = 0.002$. n = 12 from 4 mice (Triple repeat within each mouse). **(c1)** The RAGE level in hippocampus was detected by immunoblotting with the anti-RAGE antibody. **(c2)** The relative intensity was shown as fold change to Con group. One-way ANOVA followed by Tukey's test. F (3, 12) = 125.70. $^{***}P < 0.001$. n = 4 in each group. **(d1-d3)** The level of p-P38, NF-κB and cleaved caspase-3 were detected by immunoblotting with the anti-p-P38, NF-κB and cleaved caspase-3 antibodies respectively. **(d4)** Optical density value was evaluated. Data were shown as fold change to Con group. One-way ANOVA followed by Tukey's test. F (3, 8) = 160.30 (p-P38), 22.77 (NF-κB) and 88.88 (cleaved caspase-3) respectively. $^{***}P < 0.001$, $^{**}P < 0.01$. $P = 0.0018$ (Con compared with DM), $P = 0.0014$ (DM compared

with FPS) for NF-κB. n = 3 in each group. **(e1)** P-p65 level in hippocampus was assessed and was presented. **(e2)** The relative intensity of P-p65/p65 was analyzed with One-way ANOVA followed by Tukey's test. $F (3, 12) = 215.80$. $^{***}P < 0.001$. n = 4 in each group.

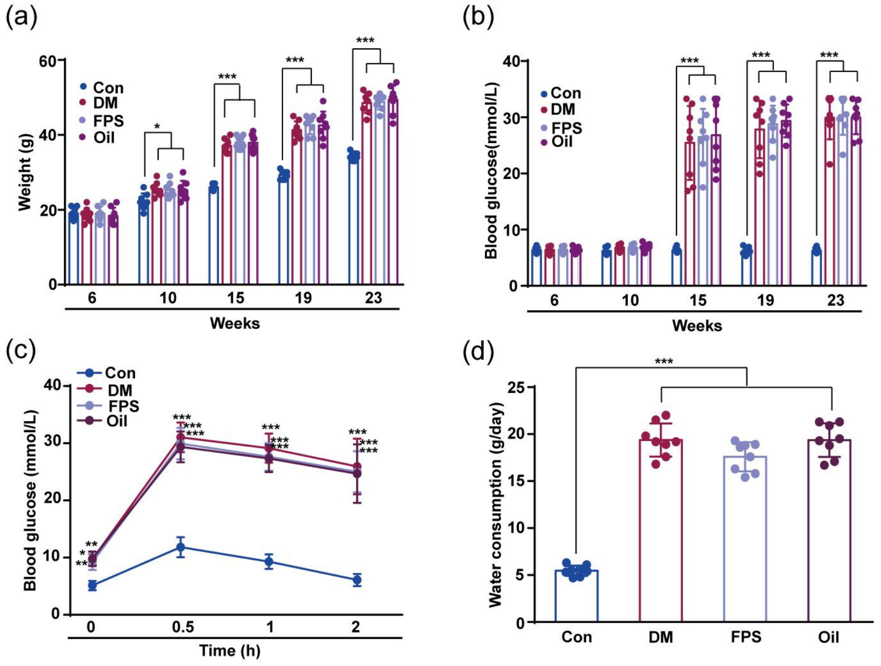

Supplementary Figure 3 FPS-ZM1 has no effect on weight, blood glucose, glucose tolerance, or water intake in STZ-induced diabetic mice. (a) Weight across different weeks were represented. Data were analyzed by two-way ANOVA and repeated measures followed by Tukey's test. In the 10th week, DM group, FPS group and Oil group compared with the Con group, P = 0.019, 0.035 and 0.035 respectively. q (8, 140) = 4.19 (Con vs DM), 3.86 (Con vs FPS) and 3.86 (Con/Oil) respectively. In the 15th, 19th, and 20th ^{weeks}, DM group, FPS group and Oil group compared with Con group, $P < 0.001$. q (8, 140) = 14.33 (Con vs DM), 15.46 (Con vs FPS) and 15.30 (Con vs Oil) in the 15th week; q (8, 140) = 15.47 (Con vs DM), 17.23 (Con vs FPS) and 17.07 (Con vs Oil) in the 19th week; q (8, 140) = 18.19 (Con vs DM), 18.52 (Con vs FPS) and 19.32 (Con vs Oil) in the 23th weekrespectively. $^{*}P <$ 0.05 and $^{***}P < 0.001$. n = 8 in each group. **(b)** Blood glucose in different groups was also analyzed by two-way ANOVA and repeated measures followed by Tukey's test. DM group, FPS group and Oil group compared with Con group, $P < 0.001$. q (8, 140) =17.70 (Con vs DM), 18.66 (Con vs FPS), and 18.98 (Con vs Oil) in the 15th week; q (8, 140) = 20.02 (Con vs DM), 20.93 (Con vs FPS), and 21.43 (Con vs Oil) in the 19th week; q (8, 140) = 21.88 (Con vs DM), 22.14 (Con vs FPS) and 21.93 (Con vs Oil) in the 23th week respectively. $^{***}P < 0.001$. n = 8 in each group. **(c)** Blood glucose levels

from 0 to 2 h during an oral glucose tolerance test. Data were analyzed by two-way ANOVA and repeated measures followed by Tukey's test. At 0 h, DM group compared with Con group, $P = 0.0045$; FPS group and Oil group compared with Con group, $P = 0.0129$ and 0.0034 respectively. At 0.5 h, 1 h, and 2 h, DM group, FPS group and Oil group compared with Con group, $P < 0.001$. q (8, 112) = 4.86 (Con vs DM), 4.38 (Con vs FPS) and 4.982 (Con vs Oil) at 0 h; q (8, 112) = 20.47 (Con vs DM), 19.30 (Con vs FPS) and 18.70 (Con vs Oil) at 0.5 h; q (8, 112) = 21.14 (Con vs DM), 20.14 (Con vs FPS) and 21.14 (Con vs Oil) at 1 h; q (8, 112) = 19.51 (Con vs DM), 19.21 (Con vs FPS) and 18.70 (Con vs Oil) at 0.5 h; q (8, 112) = 21.14 (Con vs DM), 20.14 (Con vs FPS) and 19.79 (Con vs Oil) at 2 h. $^{*}P < 0.05$; $^{**}P < 0.01$; $^{***}P < 0.001$. n = 8 in each group. **(d)** Water consumption were presented. One-way ANOVA followed by Tukey's test were used. F (3, 28) = 159.90. $^{***}P < 0.001$. n = 8 in each group.

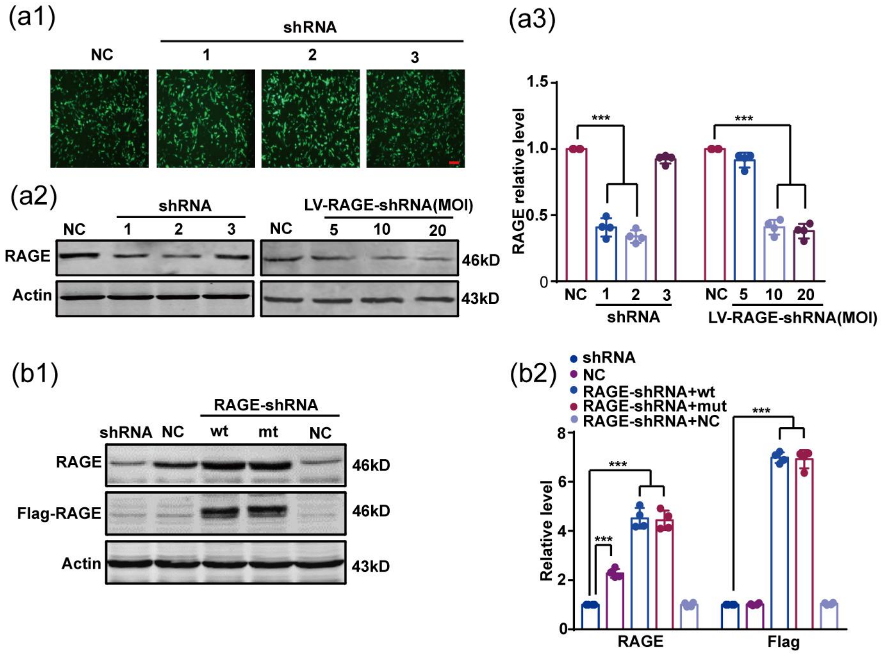

Supplementary Fig 4 RAGE knockdown in HT22 cells. (a1) Typical fluorescent images of RAGE-shRNA plasmid transfection in HT22 cell line. Scale bar is 50 µm (magnification × 100). NC: Nonsense control group, 1, 2, and 3 are different numbering of RAGE shRNAs. **(a2)** The RAGE expression in HT22 cells was tested by immunoblotting with the ant-RAGE antibody. 5. 10 and 20 are different MOI of LV-RAGE-shRNA (shRNA 2). The representative blots showed RAGE knockdown (left) and the effect of different MOI of LV-RAGE-shRNA on RAGE expression (right). **(a3)** Relative intensity was evaluated using optical density and shown as fold change to NC group. One-way ANOVA followed by Tukey's test. F (3, 12) =184.80 (left) and 232.00 (right). $^{***}P < 0.001$. n = 4 in each group. **(b1)** The Flag-RAGE over-expression in RAGE knockdown HT22 cells was evaluated by immunoblotting with the anti-RAGE and Flag antibodies respectively. **(b2)** Intensity of the RAGE and Flag were displayed as fold change to shRNA group, and were analyzed by one-way ANOVA followed by Tukey's test. F (4, 15) = 167.70 (RAGE) and 1115.00 (Flag) respectively. $^{***}P < 0.001$, n = 4 in each group.

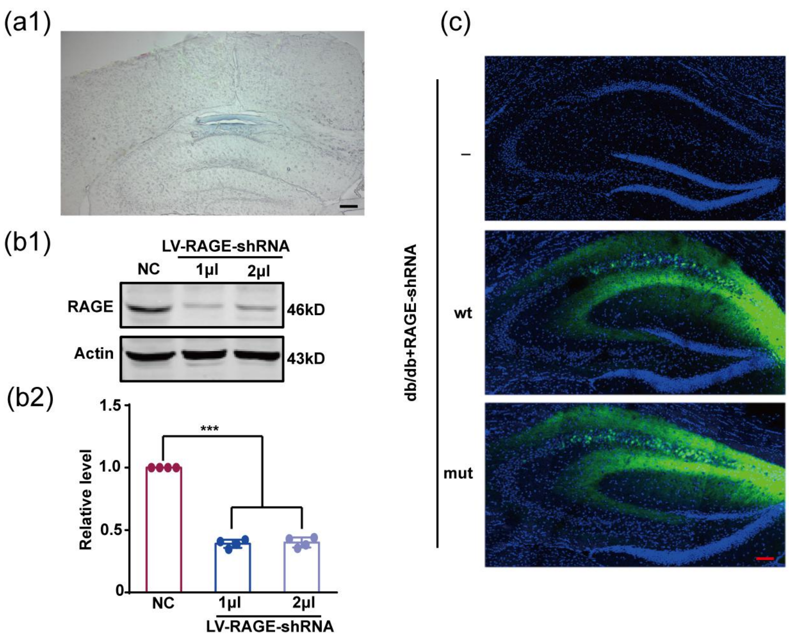

Supplementary Figure 5 RAGE knockdown in db/db hippocampus NC: Nonsense control group; 1 μl and 2 μl are different dose of LV-RAGE-shRNA. (a) The needle passage in the hippocampal CA1 subregion was marked with bromophenol blue. Scale bar is 200 μm (magnification × 100). **(b1)** Representative bands showed the effect of LV-RAGE-shRNA on RAGE level in hippocampus. **(b2)** RAGE relative level was shown as fold change to NC group. Data were analyzed by One-way ANOVA and Tukey's test. $F(2, 9) = 543.00$. $^{***}P < 0.001$. n = 4 in each group. **(c)** Typical fluorescent images showed GFP-labelled wild type and mutant LV-RAGE over-expression in hippocampus CA1 subregion. Scale bar is 200 μm (magnification × 100).

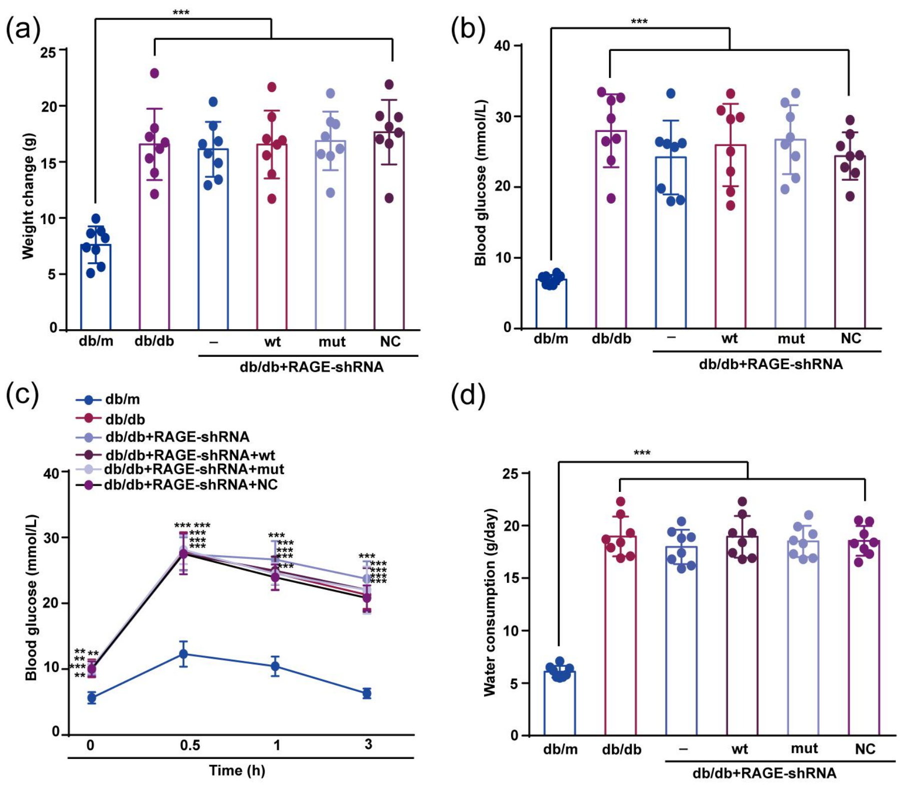

Supplementary Figure 6 Over-expression of RAGE mutation has no effect on weight change, blood glucose, glucose tolerance, or water intake in db/db mice. (a) Weight change were represented. One-way ANOVA followed by Tukey's test. F (5, 42) = 15.80. $^{***}P < 0.001$. n = 8 in each group. **(b)** Blood glucose levels in different groups were analyzed by one-way ANOVA followed by Tukey's test. F (5, 42) = 24.07, $^{***}P < 0.001$. n = 8 in each group. **(c)** Blood glucose levels from 0 to 2 h during the OGTT were analyzed with two-way ANOVA and repeated measures followed by Tukey's test. At 0 h, db/db group, db/db+RAGE-shRNA group, db/db+RAGE-shRNA+wt group, db/db+RAGE-shRNA+mut, and db/db+RAGE-shRNA+NC compared with the db/m group, P = 0.0011, 0.0021, 0.0013, 0.0005, and 0.0013 respectively; At other times, the db/db group, db/db+RAGE-shRNA group, db/db+RAGE-shRNA+wt group, db/db+RAGE-shRNA+mut, and db/db+RAGE-shRNA+NC compared with db/m group, $P < 0.001$. q (8, 168) = 5.74 (db/m vs db/db), 5.48 (db/db+RAGE-shRNA vs db/m), 5.66 (db/db+RAGE-shRNA+wt vs db/m), 5.98 (db/db+RAGE-shRNA+mut vs db/m), and 5.66 (db/db+RAGE-shRNA+NC vs db/m) at 0 h; q (8, 168) = 20.07 (db/m

vs db/db), 19.52 (db/db+RAGE-shRNA vs db/m), 19.54 (db/db+RAGE-shRNA+wt vs db/m), 20.34 (db/db+RAGE-shRNA+mut vs db/m), and 19.54 (db/db+RAGE-shRNA+NC vs db/m) at 0.5 h; q (8, 168) = 18.20 (db/m vs db/db), 20.84 (db/db+RAGE-shRNA vs db/m), 18.67 (db/db+RAGE-shRNA+wt vs db/m), 18.07 (db/db+RAGE-shRNA+mut vs db/m), and 17.38 (db/db+RAGE-shRNA+NC vs db/m) at 1 h; q (8, 168) = 19.28 (db/m vs db/db), 22.38 (db/db+RAGE-shRNA vs db/m), 20.23 (db/db+RAGE-shRNA+wt vs db/m), 20.18 (db/db+RAGE-shRNA+mut vs db/m), and 18.65 (db/db+RAGE-shRNA+NC vs db/m) at 1 h; $^{**}P < 0.01$; $^{***}P < 0.001$. n = 8 in each group. **(d)** Water consumption were displayed. Data were analyzed with one-way ANOVA followed by Tukey's test. F (5, 42) = 85.17. $^{***}P < 0.001$. n = 8 in each group.

Supplementary Table 1 All antibodies, chemicals, recombinant proteins, critical commercial assays, cell lines, experimental models, oligonucleotides and recombinant DNA used in the present work.

Antibodies		
Mouse Anti-RAGE	Santa Cruz Biotechnology	sc-365154
Rabbit Anti-MEKK3	Proteintech	13898-1-AP
Rabbit Anti-MKK3	Cell Signaling Technology	8535
Rabbit Anti-p38MAPK	Cell Signaling Technology	8690
Rabbit Anti-CCM2	Proteintech	26270
Mouse Anti-GST	Proteintech	10000-0-AP
Rabbit Anti-His	Proteintech	66005-1-Ig
Mouse Anti-Flag	Proteintech	66008-3-Ig
Rabbit Anti-Flag	Proteintech	20543-1-AP
Mouse Anti-IgG	Proteintech	B900620
Rabbit Anti-IgG	Proteintech	10285-1-AP
Mouse Anti-β-Actin	Cell Signaling Technology	3700
Rabbit Anti-β-Actin	Cell Signaling Technology	4970
Mouse Cell Signaling Technology	Cell Signaling Technology	14269
Rabbit Anti-LamimB	Proteintech	12987-1-AP
Anti-P-p38(Thr180/Tyr182)	Cell Signaling Technology	4511
Rabbit Anti-p65	Cell Signaling Technology	8242
Rabbit Anti-P-p65	Cell Signaling Technology	3033
Rabbit Anti-caspase 3	Proteintech	19677-1-AP
Mouse Anti-NeuN	Cell Signaling Technology	12943s
Rabbit Anti-Cleaved caspase 3	Cell Signaling Technology	9664
Goat anti-rabbit Alexa Fluor 488	Invitrogen	VA1022
Goat anti-mouse Alexa Fluor 488	Invitrogen	A11034
Goat anti-mouse Alexa Fluor 594	Invitrogen	A11032

Chemicals and Recombinant Proteins		
STZ	Sigma-Aldrich, St. Louis	S0130
FPS-ZM1	MedChemExpress	945714-67-0
TransInTM EL Transfection Reagent	Beijing TransGen Biotech	FT201-01
Protein A/G-Agarose	MedChemExpress	HY-K0202

Critical Commercial Assays		
ELISA Kit for AGEs	Cloud-Clone Corp	CEB353Ge
ELISA Kit for sRAGE	Cloud-Clone Corp	SEA645Mu
Annexin V-PE/7-AAD Apoptosis Detection Kit	BD Bioscience	559763
Cytoplasmic Protein Extraction Kit	keygentec	KGA826
Nucleoprotein Extraction Kit	keygentec	KGP150

Golgi Stain Kit	Fdneurotech	PK401A
PierceTM GST Protein Interaction Pull-Down Kit	Thermo Scientific	TJ2276008

Cell Lines and Experimental Models	
HEK-293T	Gift from Xuzhou Medical University Public Experimental Research Center
HT22	Gift from Xuzhou Medical University Public Experimental Research Center
**Experimental Models: Organisms/Strains**	
Mouse: C57BL/6	Xuzhou Medical University Laboratory Animal Center
Mouse: db/db and db/m	Model Animal Research Center of Nanjing University

Oligonucleotides	
RAGE-shRNA1: GCAGCTAGAATGGAAACTGAATTCAAGAGATTCAGTTTCCATTCTAGCTGCTT	SANGON
RAGE-shRNA2: TGGCAAAGAAACACTCGTGAATTCAAGAGATTCACGAGTGTTTCTTTGCCATT	SANGON
RAGE-shRNA3: GAGCTGAATCAGTCAGAGGAATTCAAGAGATTCCTCTGACTGATTCAGCTCTT	SANGON
MKK3-shRNA1: CCGGCCCATTCTTCACCTTGCACAACTCGAGTTGTGCAAGGTGAAGAATGGGTTTTT	SANGON
MKK3-shRNA2: CCGGGCTGATGGAACACCCATTCTTCTCGAGAAGAATGGGTGTTCCATCAGCTTTTT	SANGON
NC: GTTCTCCGAACGTGTCACGTCAAGAGATTACGTGACACGTTCGGAGAATT	SANGON

Recombinant DNA	
Plasmid PGEX-4T-1-GST-RAGE	SANGON
Plasmid PGEX-4T-1-GST-RAGE R362A/K363A/R364A/Q365A	SANGON
Plasmid PGEX-4T-1-GST-RAGE R383A/384A/R385A	SANGON
Plasmid PGEX-4T-1-GST	SANGON
Plasmid pcDNA3.1-His-MEKK3	SANGON
Plasmid pcDNA3.1-His-MKK3	SANGON
Plasmid pcDNA3.1-His-p38	SANGON
Plasmid pcDNA3.1-His-OSM	SANGON
Plasmid pcDNA3.1-His	SANGON

Lentivirus hU6-MCS-CMV-Puromycin-MKK3-shRNA1	GeneChem
Lentivirus hU6-MCS-CMV-Puromycin-RAGE-shRNA2	GeneChem
Lentivirus hU6-MCS-CMV-Puromycin-NC	GeneChem
Lentivirus Ubi-MCS-3FLAG-SV40-Neomycin-RAGE	GeneChem
Lentivirus Ubi-MCS-3FLAG-SV40-Neomycin-RAGE R352/K353/R354/Q355A	GeneChem
Lentivirus Ubi-MCS-3FLAG-SV40-Neomycin-NC	GeneChem
